# What brain abnormalities can magnetic resonance imaging detect in foetal and early neonatal spina bifida: a systematic review

**DOI:** 10.1007/s00234-021-02853-1

**Published:** 2021-11-18

**Authors:** Nada Mufti, Adalina Sacco, Michael Aertsen, Fred Ushakov, Sebastian Ourselin, Dominic Thomson, Jan Deprest, Andrew Melbourne, Anna L. David

**Affiliations:** 1 Elizabeth Garrett Anderson Institute for Women’s Health, University College London, London, UK; 2 School of Biomedical Engineering and Imaging Sciences (BMEIS), King’s College London, London, UK; 3 Fetal Medicine Unit, University College London Hospital NHS Foundation Trust, London, UK; 4 Department of Radiology, University Hospitals Katholieke Universiteit (KU) Leuven, Leuven, Belgium; 5 Paediatric Neurosurgery Department, Great Ormond Street Hospital for Children, London, UK; 6 Department of Obstetrics and Gynaecology, University Hospitals Katholieke Universiteit (KU) Leuven, Leuven, Belgium

**Keywords:** Spina bifida, Intracranial anomaly, Foetal surgery, Systematic review, MRI

## Abstract

**Purpose:**

Open spina bifida (OSB) encompasses a wide spectrum of intracranial abnormalities. With foetal surgery as a new treatment option, robust intracranial imaging is important for comprehensive preoperative evaluation and prognostication. We aimed to determine the incidence of infratentorial and supratentorial findings detected by magnetic resonance imaging (MRI) alone and MRI compared to ultrasound.

**Methods:**

Two systematic reviews comparing MRI to ultrasound and MRI alone were conducted on MEDLINE, EMBASE, and Cochrane databases identifying studies of foetal OSB from 2000 to 2020. Intracranial imaging findings were analysed at ≤ 26 or > 26 weeks gestation and neonates (≤ 28 days). Data was independently extracted by two reviewers and meta-analysis was performed where possible.

**Results:**

Thirty-six studies reported brain abnormalities detected by MRI alone in patients who previously had an ultrasound. Callosal dysgenesis was identified in 4/29 cases (2 foetuses ≤ 26 weeks, 1 foetus under any gestation, and 1 neonate ≤ 28 days) (15.1%, CI:5.7–34.3%). Heterotopia was identified in 7/40 foetuses ≤ 26 weeks (19.8%, CI:7.7–42.2%), 9/36 foetuses > 26 weeks (25.3%, CI:13.7–41.9%), and 64/250 neonates ≤ 28 days (26.9%, CI:15.3–42.8%). Additional abnormalities included aberrant cortical folding and other Chiari II malformation findings such as lower cervicomedullary kink level, tectal beaking, and hypoplastic tentorium. Eight studies compared MRI directly to ultrasound, but due to reporting inconsistencies, it was not possible to meta-analyse.

**Conclusion:**

MRI is able to detect anomalies hitherto underestimated in foetal OSB which may be important for case selection. In view of increasing prenatal OSB surgery, further studies are required to assess developmental consequences of these findings.

**Supplementary Information:**

The online version contains supplementary material available at 10.1007/s00234-021-02853-1.

## Introduction

Open spina bifida (OSB) is a non-lethal condition with complex physical and neurodevelopmental sequalae [1–6]. It comprises brain abnormalities which include the Chiari II malformation (CIIM), a constellation of anomalies, principally, but not exclusively associated with the brainstem and characterised by hindbrain herniation in which the medulla, fourth ventricle, and cerebellum are displaced into the spinal canal. Cerebrospinal fluid (CSF) circulation is disturbed in OSB predisposing to hydrocephalus and mechanical distortion of the brain parenchyma, damaging white matter tracts. Additional developmental anomalies of the supratentorial brain are increasingly recognised in OSB including heterotopia and callosal dysgenesis [1, 7–12].

Foetal OSB surgery is now offered due to level 1 evidence that prenatal closure reduces ventriculoperitoneal shunt requirement by 40% and hindbrain herniation at 12 months [1, 7]. Before embarking on surgery, it is important to comprehensively assess the foetal brain with robust imaging to provide optimal parental counselling, as it is invasive with maternal morbidity [13]. Although ultrasonography is the primary imaging modality, it is susceptible to poor intracranial images due to factors such as maternal habitus, foetal position, and reverberation artefacts from bones of the calvarium[2, 14–16]. MRI, conversely, provides excellent contrast and spatial resolution, permitting detailed anatomical evaluation of the posterior fossae and brain parenchyma[15–17]. However, as surgery is performed before 25 + 6 weeks gestation, assessment must be carried out in the early second trimester[1], posing challenges due to small foetal size and increased foetal motion, both of which affect MRI spatial resolution [18]. To mitigate this, advances such as rapid MR imaging have been introduced to reduce imaging time and provide acceptable resolution as early as 18 weeks gestation [19, 20]. We sought to determine MRI value in addition to ultrasound in intracranial assessment of OSB foetuses in the context of foetal surgery. Two systematic reviews were conducted to determine the incidence of typical (widely accepted features that occur in spina bifida) and additional infratentorial, supratentorial, and miscellaneous intracranial findings detected by conventional and advanced MRI in comparison with ultrasound (systematic review 1 (SR1)) and without direct ultrasound comparison (systematic review 2, SR2) at ≤ 26 weeks GA, > 26 weeks GA, and the early neonatal period, ≤ 28 days. Reported infratentorial findings will be in reference to structural abnormalities in the lower region of the brain below the tentorium cerebelli (e.g. the CIIM), while supratentorial findings are in relation to anomalies overlying the tentorium in the upper part of the brain (e.g. ventricular, cerebrospinal fluid, and cortical malformations). Miscellaneous findings pertain to all other anomalies such as intracranial haemorrhages and cysts.

## Materials and methods

### Funding

This work is funded by the Wellcome Trust [203148/Z/16/Z;203145Z/16/Z; WT101957] and Engineering and Physical Sciences Research Council [NS/A000049/1; NS/A000050/1; NS/A000027/1; EP/L016478/1]. This grant included external peer review for scientific quality with a patient and public involvement panel. The funders had no direction in the study design, data collection, data analysis, manuscript preparation, or publication decision.

### Protocol and registration

Two systematic reviews were performed according to the Preferred Reporting Items for Systematic reviews and Meta-analyses (PRISMA) guidance [21]. Both protocols were registered with the International Prospective Register of Systematic Reviews (CRD42019124966 and CRD42020167567).

### Search strategy

We conducted two systematic reviews on MEDLINE, EMBASE, and Cochrane databases using a combination of medical subject headings and keywords (Online Resource: Search Strategy). In order to identify ‘grey’ literature, the first 100 results in PubMed and Google Scholar were screened, and reference lists of review articles and eligible papers were searched to retrieve relevant additional articles. Elimination of duplicates and management of study screening was performed using the Covidence software (Veritas Health Innovation Ltd, Melbourne, Australia).

### Study selection

Two authors (NM and AS) independently screened all titles and abstracts and excluded irrelevant studies. The remaining full-text articles were then independently assessed for eligibility. Any disagreements were resolved by consensus.

### Eligibility criteria

All randomised, case–control, cross-sectional, cohort studies, case series, and case reports reporting brain abnormalities using MRI in comparison to ultrasound (SR1) or without direct ultrasound comparison (MRI alone, SR2) from 2000 to 2020 were included. No language restrictions were applied. Systematic reviews and narrative review articles were excluded. We included human studies involving spina bifida in the foetal and early neonatal period (≤ 28 days). Post-mortem examination studies were excluded. We included both open and closed spina bifida studies, as some have directly compared MRI findings between these two distinct diseases to highlight the lack of intracranial sequalae seen in closed spina bifida foetuses in comparison, which is important in clinical screening and diagnosis. Studies were excluded if other intracranial abnormalities such as anencephaly or chromosomal abnormalities were reported, and it was not possible to selectively extract relevant data if results were collectively reported with other patients without genetic anomalies. Studies assessing only the foetal spine were also excluded. Studies from which data could not be extracted due to combined outcomes reported for a range of intracranial anomalies were also excluded.

### Data extraction

Two authors (NM and AS) independently extracted data which was entered into a pre-piloted Excel (Microsoft, Washington, USA) form. Disagreements were resolved by consensus. Recorded study characteristics included study design and presence of control group. The type of surgery (e.g. postnatal, open foetal surgery, or foetoscopy) was noted. The population was split into foetuses ≤ 26 weeks GA, > 26 weeks GA, early neonatal period (≤ 28 days), and ‘all gestations’ if GA at scan was not specified. A cut-off of 26 weeks GA was chosen, as this is the latest GA when foetal surgery shows evidence of efficacy [1, 7]. The number of subjects was noted in each study, as well as the number of scans at each time point, taking into account foetuses undergoing longitudinal scanning due to foetal surgery. In SR1, the type of ultrasound used was noted (2D, 3D, or 4D), and in both reviews, the type of MRI was recorded to capture traditional (e.g. single-shot fast spin echo (SSFSE)) and advanced sequences (e.g. diffusion-weighted imaging (DWI)). Intracranial abnormalities were separated into typical or frequent infratentorial (hindbrain herniation and cerebellar abnormalities) and supratentorial (hydrocephalus/ventriculomegaly, callosal dysgenesis, and heterotopias) findings. Additional intracranial abnormalities were also noted across MRI studies. Typical post-operative features such as reversal of CIIM (complete or partial) and stable, reduced, or increased ventriculomegaly were also recorded, as were any additional intracranial features after foetal surgery. Postnatal confirmation of intracranial abnormalities was likewise recorded (including post-mortem MRI in the event of termination of pregnancy after antenatal diagnosis).

### Quality assessment (QA)

Two authors (NM and AS) assessed study quality and risk of bias independently. In the inclusion criteria, all types of randomised control trials and observational studies (e.g. case–control, cross-sectional, cohort studies, and case series and case reports) were initially included, with the exclusion of systematic reviews and review articles. There were only two types of studies eventually extracted after the screening and eligibility assessment stages: case–control and case series. The Newcastle–Ottawa scale was used for assessing quality of case–control studies rating bias according to case selection, comparability, and exposure [22]. The National Institutes of Health study quality assessment tool was used for assessing risk of bias in case series [23].

### Reporting of findings

Meta-analysis for typically reported infra and supratentorial brain abnormalities and commonly reported post foetal closure operative findings were carried out using Comprehensive Meta-Analysis version 3 software (Biostat, Inc., Englewood, USA). Results were expressed as rates with 95% confidence intervals (CI). Pooled rates were calculated using the random effects model due to clinical and statistical heterogeneity between studies. No meta-analysis was performed on additional intracranial abnormalities recorded using either traditional or advanced MRI and post-operative intracranial features due to the wide range reported. The incidence of these findings was expressed as either a percentage (number of specific intracranial abnormality/total patients for each study × 100) or as the *p*-value reported in studies if numbers of the abnormality was not provided.

### Assessment of heterogeneity

Methodological and clinical heterogeneity of each study was evaluated. Wherever data was pooled, variables were tested for statistical heterogeneity using the *I*^2^ test. An *I*^2^ value of less than 40%, 40–75%, and > 75% indicated minor, moderate, and substantial heterogeneity, respectively[24].

## Results

### Study selection

**Fig. 1:**
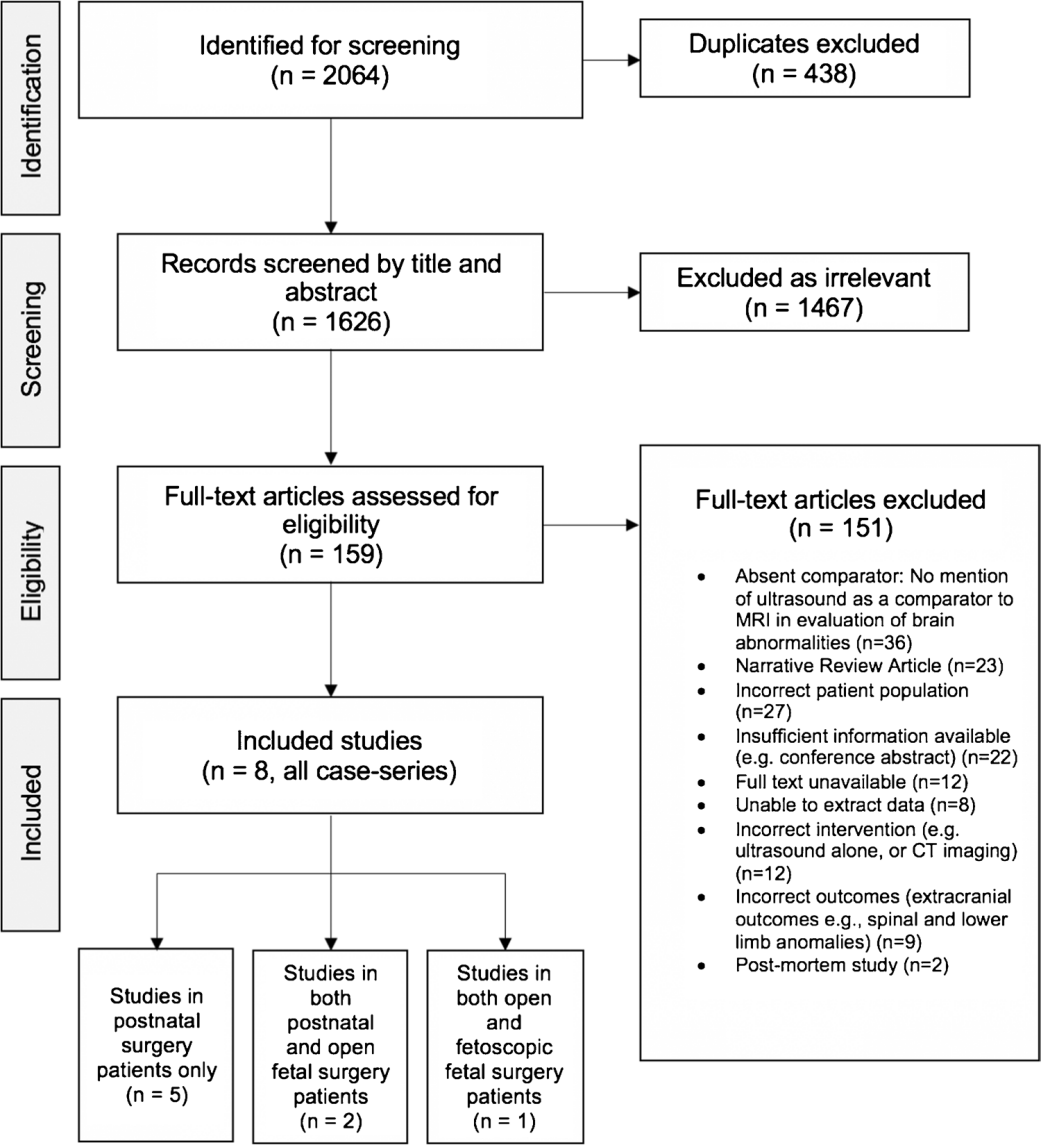
Flow diagram for study selection of systematic review comparing MRI to ultrasound in detection of additional brain abnormalities in foetal and early neonatal spina bifida. Adapted from PRISMA 2008 [25]

The electronic search for SR1 (MRI directly compared to ultrasound) identified 2055 studies (Fig. 1). A further 9 studies were identified by searching ‘grey’ literature and reference lists. Following this, 438 studies were excluded as duplicates. The remaining 1626 studies were screened by abstract and title, of which 1467 were excluded as irrelevant. Full texts of the remaining 159 studies were reviewed, and 151 were excluded for reasons such as failure to mention ultrasound in direct comparison to MRI (36/151, 23.8%). Such studies were included in SR2 instead. Eventually, 8 studies were included which did not include ‘grey’ literature studies.

**Fig. 2:**
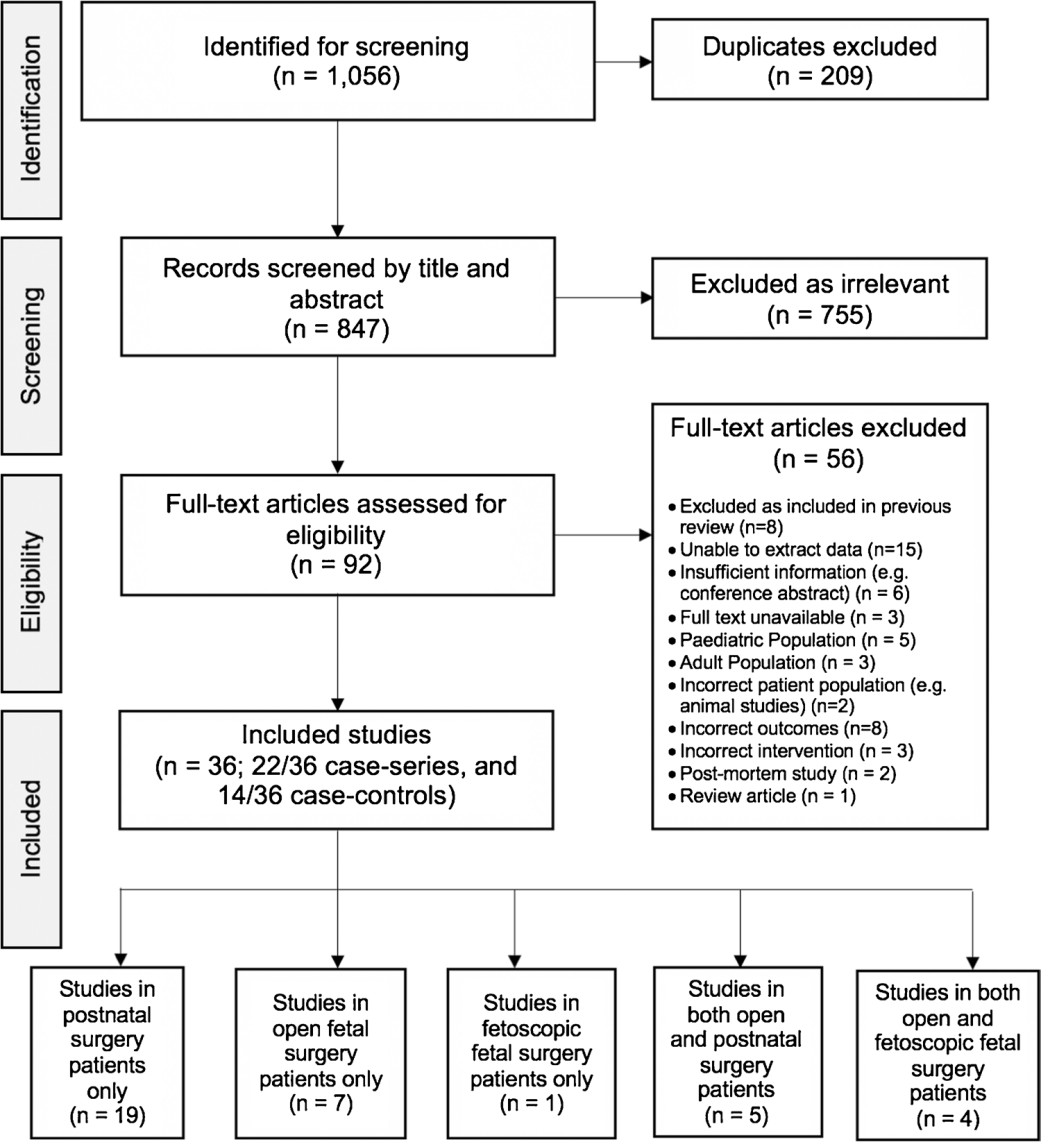
Flow diagram for study selection of systematic review on detection of brain abnormalities by MRI in foetuses diagnosed with OSB by ultrasound. Adapted from PRISMA 2008 [25]

In SR2 (MRI in foetuses diagnosed with OSB by ultrasound), 1497 results were identified (Fig. 2). Further 9 studies were identified from ‘grey’ literature and reference lists after which 209 studies were removed as duplicates. The remaining 847 studies were screened by title and abstract, of which 755 were irrelevant. Full texts of the remaining 92 articles were reviewed, and 56 were excluded for reasons such as inability to extract data due to combined outcomes reported across a range of foetal intracranial abnormalities (15/56, 26.8%). Eventually, 36 studies were selected, including 4 studies from the ‘grey’ literature [26–29]. There was only one non-English study which was translated from Polish.

### Characteristics and quality of studies

**Fig. 3:**
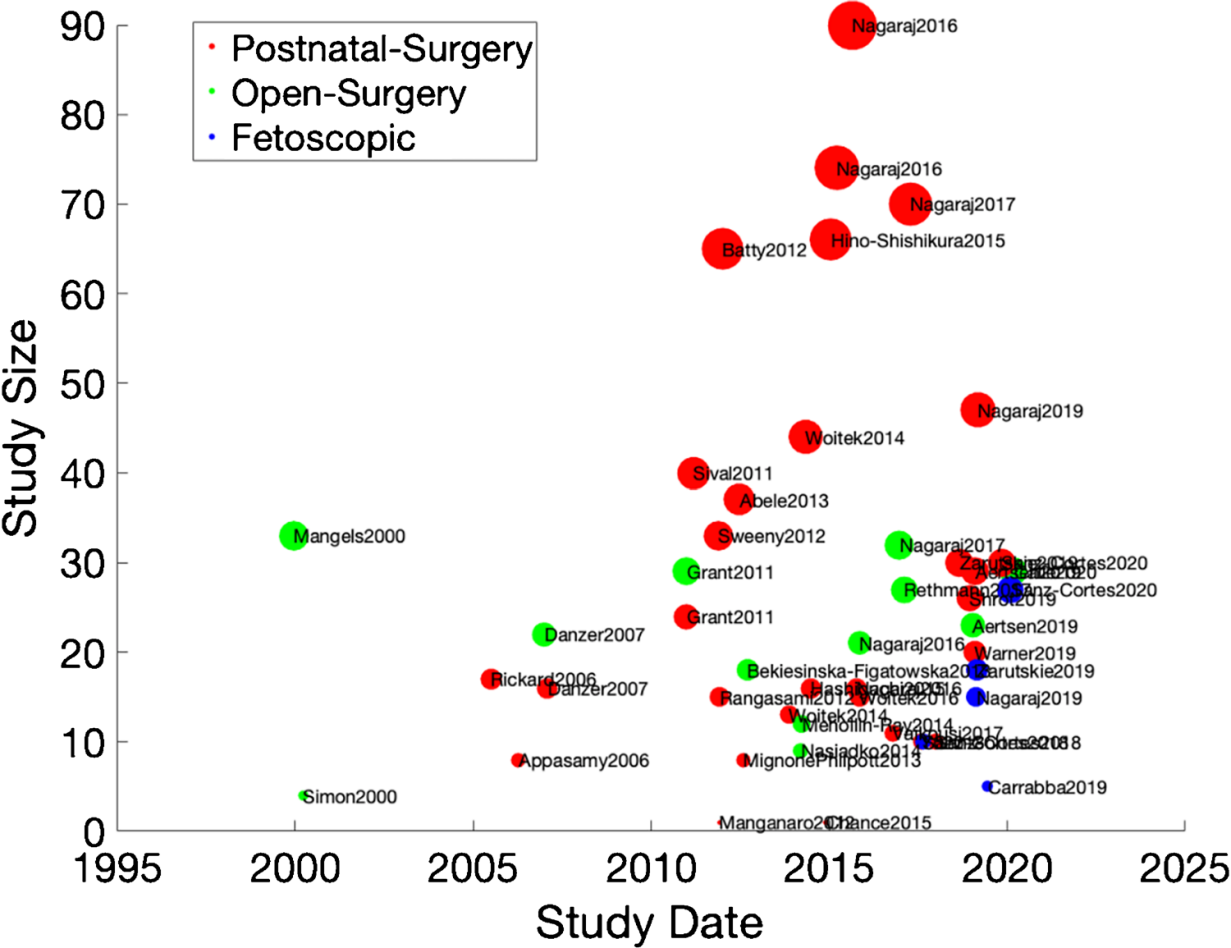
Graphical timeline display of all studies in both systematic reviews according to spina bifida surgery type

Characteristics of included studies in SR1 and SR2 are shown in Online Resource Tables 1 and 2. Types of surgeries across both reviews were (24) postnatal, (7) open foetal surgery, (1) foetoscopic, (7) open and postnatal, and (5) open and foetoscopic foetal surgery (Fig. 3).

**Fig. 4:**
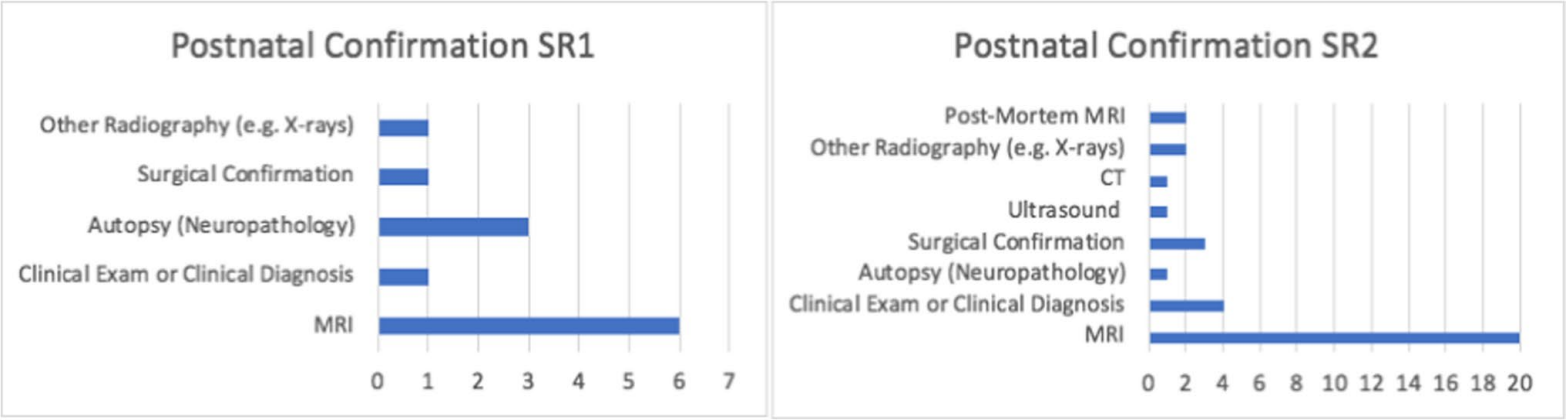
Types of postnatal confirmation in systematic review (SR) 1 (left) and SR2 (right)

Open prenatal surgery is in reference to a maternal laparotomy with hysterotomy to perform foetal closure as opposed to the foetoscopic route. MRI has been used to study both open foetal and postnatal surgeries from the late 1990s, with larger postnatal series published from 2010 and foetoscopic surgery studies available after 2015. Only studies from 2000 onwards were included, to reflect the timing of introduction of MRI fast sequences. The MRI sequences described in both reviews are shown in Tables 1 and 2 below, exhibiting the acquisition of both advanced and traditional sequences worldwide in the foetal and neonatal settings. Types of postnatal confirmation of intracranial abnormalities are also shown in Fig. 4 below.

**Table 1:** MRI and sequences in systematic review 1

MRI type/sequence	*N* = number of different types of MRI	*N*/number of Studies = (*N*/8) %
Foetal and neonatal studies		
HASTE (half-Fourier acquisition single-shot turbo spin echo)	3	37.5
Not stated	4	50.0
GRE (gradient echo)	1	12.5
DWI (diffusion-weighted imaging)	1	12.5
Foetal studies only		
RARE (rapid acceleration with relaxation enhancement)	1	12.5
Single-shot high resolution (SSH)	1	12.5
Balanced field echo	1	12.5

**Table 2:** MRI and sequences in systematic review 2

MRI type/sequence	*N* = number of different types of MRI	*N*/number of studies = (*N*/36) %
Foetal and neonatal studies		
HASTE (half-Fourier acquisition single-shot turbo spin echo)	8	22.2
SSFSE (single-shot fast spin echo)	14	38.9
SSFP (steady-state free precession)	3	8.3
T1W (T1 weighted)	5	13.9
EPI (echo planar imaging)	3	8.3
GRE (gradient echo)	2	5.5
DWI (diffusion-weighted imaging)	9	25
Foetal studies only		
DTI (diffusion tensor imaging)	1	2.8
RARE (rapid acceleration with relaxation enhancement)	1	2.8
FIESTA (fast imaging employing steady-state acquisition)	1	2.8
SWI (susceptibility-weighted imaging), e.g. BOLD	1	2.8
Neonatal studies only		
3 T (3 Tesla)	1	2.8
FLAIR (fluid attenuated inversion recovery)	1	2.8

QA of the studies are given in Online Resource Figs. 1, 2, and 3. In SR1, all studies were case series, with either a ‘good’ (7/8, 87.5%) or ‘fair’ (1/8, 12.5%) overall risk of bias score. In SR2, 22/36 or 61.1% of studies were case series, with an overall risk of bias score of ‘good’ (13/22, 59.1%), ‘fair’ (8/22, 36.3%), and ‘poor’ (1/22, 4.5%). For both reviews, all domains of QA of case series scored ‘high quality’, apart from one quality measure involving description of statistical methods. In SR2, 14/36 or 38.9% of studies were case-controls, with either ‘good’ (13/14, 92.9%) or ‘fair’ (1/14, 7.1%) overall risk of bias score. The risk of bias in all domains for QA of case–control studies was ‘high quality’, apart from one quality measure involving selection of controls as they are all hospital controls (14/14, 100%).

### Statistical heterogeneity

Brain abnormality data was pooled into 21 separate meta-analyses, of which 47.6% (10/21) had no or minor heterogeneity, 23.8% (5/21) had moderate, and 28.6% (6/21) had considerable heterogeneity. Intracranial post-operative findings were pooled into 9 separate meta-analysis, of which 44.4% (4/9) had minor or no heterogeneity, 22.2% (2/9) had moderate, and 33.3% (3/9) had considerable heterogeneity. A more detailed breakdown can be found in Online Resource Tables 3 and 4.

### Direct comparison between USS and MRI, SR1

Due to high clinical diversity and statistical heterogeneity in SR1, meta-analysis and direct comparison of ultrasound to MRI were not performed. Diversities included a wide range of intracranial abnormalities reported across a small number of studies. Other clinical diversities included little or no description of ultrasound route (i.e. transabdominal and/or transvaginal), MRI protocols and sequences not stated, gestational and neonatal age not specified at time of MRI, minimal detail on interval time between ultrasound and MRI, and sparse detail on operator expertise. Furthermore, imaging quality of provided images in the extracted studies in SR1 (*n* = 8) was subjectively assessed based on contrast, spatial resolution, and presence of artefact. This showed less than optimal image quality in 7 out of 8 studies. Quantitative assessment was not possible as certain parameters (e.g. pixel spacing, echo, and repetition times) were not provided. Given these heterogeneities, studies extracted in SR1 were not included in SR2. Some features identified by MRI over ultrasound included diagnosis of corpus callosum agenesis and a ‘tight posterior fossae’, whereby the cerebrospinal fluid (CSF) signal was absent or barely visible [30]. Levine et al. additionally described more frequent detection of an angular configuration of the frontal and occipital margins of lateral ventricles by MRI in comparison to ultrasound which can serve as secondary sign of the presence of OSB [15]. Maurice et al. reported additional findings of perinodular heterotopia in two cases using MRI, whereas other studies demonstrated moderate agreement between the two imaging modalities in reporting heterotopia [31, 32]. Araujo Junior et al.[2] confirmed concordance between ultrasound and MRI in the assessment of the size of the atrium of the lateral ventricle and cerebellar shortening percentage. This was performed to compare ultrasound and MRI in assessment of brain parameters in foetuses with OSB.

### Typical or frequent brain abnormalities and post-operative findings, SR2

Meta-analysis in SR2 was performed on typical or frequent brain abnormality data and commonly reported post-operative features in OSB only (Online Resource Tables 5 and 6).

#### Typical or frequent brain abnormalities

Incidence of callosal dysgenesis in fetuses and neonates ≤ 28 days was 15.1% (CI 5.7–34.3%). Heterotopia diagnosis in foetuses ≤ 26 weeks, foetuses > 26 weeks, and neonates ≤ 28 days was 19.8% (CI 7.7–42.2%), 25.3% (CI 13.7–41.9%), and 26.9% (CI 15.3–42.8%), respectively.

#### Post-operative findings

The presence of Chiari II malformation is one of the eligibility criteria for foetal surgery. Improvement in Chiari II malformation is now considered an objective indicator of the success of prenatal repair of spinal bifida. Confirmation of Chiari II improvement across foetuses > 26 weeks GA, neonatal ≤ 28 days, and all gestations was 85.9% (CI 66.3–94.9%). With respect to ventricular size, in foetuses > 26 weeks GA, the detection rates for reduced ventriculomegaly post-operatively were 6.3% (CI 1.6–22.2%) and 76.8% (CI 63.1–86.4%) for increased ventriculomegaly post-operatively.

### Additional intracranial findings using traditional MRI techniques

Additional brain abnormalities reported using traditional MRI sequencing such as half-Fourier-acquired single-shot turbo spin echo (HASTE) and single-shot fast spin echo (SSFSE) were subdivided into infratentorial, supratentorial, and miscellaneous (Online Resource Tables 7, 8, and 9).

#### Infratentorial intracranial findings using traditional MRI techniques

Infratentorial measurements such as transverse diameter of the posterior fossae (TDPF), posterior fossae area (PFA), trans-cerebellar diameter (TCD), and clivus supra-occiput angle (CSA) were reduced in comparison to age-matched controls. These findings are thought to reflect impaired growth of the posterior fossae and its contents in response to the continuous loss of CSF through the spinal defect. This hypothesis is supported by the observation that following foetal surgery, these measures increased significantly with an evolution towards normal compared to OSB foetuses who had postnatal surgery [6, 8, 33–37]. Other detailed anatomical assessment and description of the CIIM were also possible using traditional MRI techniques, such as lower level of cervicomedullary kink and low pontomesencephalic junction [38]. These were found to be associated with malformations of cortical development which may present with symptoms such as epilepsy, developmental delay, and focal neurological signs [38]. Other CIIM findings included flattening of the fourth ventricle and tectal beaking or hypoplastic tentorium [3, 38]. This may also be important in terms of prognostication as it gives an indication as to the severity of the condition.

#### Supratentorial intracranial findings using traditional MRI techniques

Supratentorial findings included ventricular abnormalities such as colpocephaly (disproportionate enlargement of the occipital horns of the lateral ventricle) at a rate of 14/33 (42.4%) and increased third ventricle size, 70/74 (94.6%) [39, 40]. Cortical anomalies such as reduced brain thickness and polymicrogyria were also reported [37, 41, 42]. Reduction in CSF space at cerebellar width level and subarachnoid space was also noted[37, 40, 42].

#### Miscellaneous intracranial findings using traditional MRI techniques

Intraventricular haemorrhages (IVH) were additionally identified such as subependymal haemorrhages (1/4 = 25%) as shown in Online Resource Table 9. Haemorrhagic contamination of the CSF might be an exacerbating factor in the pathogenesis of hydrocephalus and persistence of hindbrain herniation [31, 43].

### Additional post-operative MRI features using traditional MRI techniques

After foetal surgery, persistent features of the CIIM were also described (Online Resource Table 10) including persistent herniation of the choroid plexus through the foramen magnum, flattening of the inferior pontine notch after birth, and continual dysplastic appearance of the tectal plate [42]. Enlargements of the in extra-axial CSF space were repeatedly seen in patients with OSB who had foetal surgery, presumed to be as a result of restoration of intracranial CSF volume. These improvements were more evident following foetal surgery than postnatal surgery.

Most authors suggest that closure of the defect promotes reversal of cerebellar herniation which reopens the communication between the fourth ventricle and subarachnoid space [37, 42, 44, 45]. The pathogenesis of hydrocephalus in OSB is however likely multifactorial and other authors have postulated that impaired absorption of CSF back into the venous system (communicating hydrocephalus) is a contributory factor. Other observations noted in patients after foetal compared to postnatal surgery included resolved aqueductal stenosis and reduction in lateral and third ventricle size [40, 42, 46].

### Additional intracranial findings using novel/advanced MRI techniques

Additional intracranial abnormalities were also noted in studies using advanced MRI sequences such as DWI, diffusion tensor imaging (DTI), and echo planar imaging (EPI) which permit quantitative evaluation of certain microstructural brain properties (Online Resource Table 11). Some examples are explained below.

#### Apparent diffusion coefficient (ADC)

Apparent diffusion coefficient (ADC) measures water molecule diffusion within tissues and was higher in the cerebellum, genu of corpus callosum, and basal ganglia of OSB patients in comparison to age-matched controls [47]. The reasons for this are unclear but may be an indirect reflection of impaired CSF drainage in the posterior fossa leading to increased extracellular water and white matter oedema[47, 48]. By contrast, frontal and temporal lobes exhibited lower ADC values compared to age-matched, normal controls which some authors speculate may be due to hydrocephalus which can lead to severe compression and ischaemic injury[49]. Although it is possible to visualise cerebellar oedema, for instance, by identification of various anatomical markers on traditional T2 sequences, it may be subjective and dependent on operator expertise. Use of diffusivity-derived parameters has the potential to provide a more quantitative approach reflecting the microstructure of the developing foetal MMC brain [47–49].

#### Fractional anisotropy

Fractional anisotropy (FA) is a marker estimated from DTI measurement, quantifying preferred directionality of water molecule motion within tissues. An increase in FA has been shown to occur in asymptomatic compressed nerve roots [50]. FA was observed to be higher in the midbrain of OSB foetuses compared to age-matched controls with normal intracranial features, which may be due to brainstem compression in the axial plane, which limits the diffusivity of water molecules axially but to a lesser extent parallel to nerve fibres [50].

#### Super resolution MRI reconstruction

Other studies demonstrated increased ventricular volume and growth after foetal surgery compared to normal age-matched controls by using superresolution reconstruction (SRR), whereby three orthogonal T2-weighted image stacks are co-registered, creating a 3D volume absent from motion artefact[48, 51]. This may be due to a lag period while CSF absorption pathways mature in response to the increased CSF load post repair.

## Discussion

### Main findings and interpretation

Ultrasound is the initial imaging modality of choice in foetal assessment due to its low cost and real-time capability. However, comprehensive intracranial assessment is not always possible due to factors such as maternal habitus and foetal position. MRI is an important adjunct due to its ability to provide enhanced visualisation through multiplanar imaging with a larger field of view. In the context of foetal OSB surgery, MRI serves three main purposes. Firstly, due to its diagnostic sensitivity, it is valuable in confirming and better characterising intracranial changes associated with OSB, secondly to screen patients eligible for foetal surgery, and thirdly to evaluate response to foetal surgery.

This review has shown that MRI identifies callosal dysgenesis and heterotopia at rates of 15.1% and 25.5% across all gestations and in the early neonatal period, respectively, in OSB patients. Heterotopia was most commonly seen in neonates ≤ 28 days, followed by foetuses > 26 weeks, and was least likely to be detected ≤ 26 weeks of gestation. This higher detection rate in the late foetal and early neonatal period could be related to foetal motion artefacts, small neuroanatomical size, and stretching of the germinal matrix due to ventriculomegaly encountered at earlier gestations [52]. Supratentorial abnormalities such as periventricular nodular heterotopia and callosal dysgenesis are related to neuronal migration impairment, triggered by altered CSF hydrodynamics in OSB [49]. Patients with these cortical developmental disorders can present with epilepsy and cognitive and developmental delay [38, 52]. The strength of correlation between these imaging findings and long-term functional outcome has yet to be defined. Nonetheless, these findings indicate that MRI has a potential prognostic role as well as guiding decision making for foetal surgery by both parents and clinicians. Although the detection rate for supratentorial abnormalities prior to 26 weeks’ gestation is low, it still consolidates the need for utilising MRI as an adjunct for preoperative foetal surgery assessment where these findings may have been otherwise challenging to detect initially with ultrasound imaging. This is important so as to improve detection rates at an earlier gestational age. Furthermore, there are promising potentials in using post-MR acquisition novel technology, such as SRR, to provide isotropic high-quality 3D images absent from foetal motion artefact with better geometric integrity which can increase identification of these cortical malformations in the future. This would be a critical area of improvement going forward for appropriate candidate selection and enhanced surgical prognostication.

Further intracranial abnormalities detected by MRI included angular ventricular appearance, cortical anomalies such as polymicrogyria, and altered CSF hydrodynamic changes such as effacement, whereby the pre-axial space is partially or completely obliterated due to lack of CSF[37, 40, 42, 53, 54]. Understanding these additional intracranial features is paramount to determine whether they are part of a wider, malformative component of OSB, with independent prognostic significance (potentially precluding foetal surgery), or whether these represent a mechanical deformation of the CNS that might be ameliorated by successful foetal surgery.

MRI is also important in assessment of brain abnormalities after foetal surgery. For example, studies observed that ventriculomegaly increased in 76.8% of foetuses imaged > 26 weeks GA. This highlights a possible need for longitudinal imaging to monitor antenatal evolution of ventricular size post-surgery. Furthermore, precise anatomical evaluation of the CIIM was made possible using MRI due to improved visualisation of anatomical boundaries. This includes findings such as a persistent dysplastic appearance of the tectal plate which is important for post-operative prognostication and management as it may raise the concern of presence of aqueductal stenosis which may be predictive of the need for ventricular shunting [1, 6, 7, 42]. These studies have also shown the potential for other measurements such as CSA, TDPF, TCD, and PFA in enhancing post-operative assessment [6, 36, 42]. Other post-operative markers of improvement included widening of supratentorial total subarachnoid space after foetal surgery [26, 42]. Furthermore, quantitative ventricular volume calculations have been made possible by MRI innovations such as SRR which mitigates foetal motion [51]. These post-operative markers collectively can serve as tools to enhance surgical efficacy evaluation which can be advantageous in assisting postnatal management such as predicting the need for postnatal hydrocephalus treatment.

This systematic review also illustrates how advanced MRI sequencing such as DWI and DTI can provide functional as well as structural information about the foetal brain in OSB. DWI can characterise brain microstructure and maturation by providing quantifiable measurements of water diffusion using ADC values [47, 48]. Restricted venous return and vasogenic oedema caused by altered CSF flow and obstruction due to the CIIM are reflected in higher ADC values in the cerebellum, basal ganglia, and genu of corpus callosum [47, 48]. Similarly, DTI provides markers such as elevated FA in the brainstem secondary to external compression due to the CIIM [50]. These measurements have the potential to provide quantifiable results which can help diagnose foetuses with OSB in the absence of clear structural imaging with the added potential of predicting CIIM severity (e.g. brain stem compression), thus enhancing prenatal surgery stratification[50].

This review confirms that foetal MRI has led to identification of intracranial abnormalities not conventionally associated with spina bifida and which may have eluded detection by first-line imaging techniques. Such findings may have implications for selection of appropriate candidates for foetal surgery. For instance, differentiation between open and closed spina bifida was achievable as a more acute CSA was seen in open versus closed spina bifida [33]. This has immense practical significance since other dysraphic malformations such as limited dorsal myeloschisis, which may appear (on antenatal imaging) to be indistinguishable from myelomeningocele, are not yet suitable for prenatal surgery. Furthermore, detection of more subtle post repair intracranial changes might also be beneficial in evaluating relative efficacy of different types of foetal surgery, such as open versus foetoscopic. For example, Sanz-Cortez et al.[48] illustrated similarly increased ADC in the genu of the corpus callosum after both open prenatal and foetoscopic repairs, demonstrating comparable intracranial findings in both techniques.

### Strength and limitations

Our data systematically reports conventional and additional intracranial features in spina bifida using traditional and advanced MRI sequencing with respect to foetal and neonatal time points. It additionally provides detail of post-operative MRI features after foetal surgery. Both aspects will be useful for counselling, selection of appropriate foetal surgery candidates, and determining surgical efficacy. One limitation of this work was that some articles did not specify gestational age at foetal MRI, so detection rates of specific abnormalities in foetuses ≤ 26 or > 26 weeks GA may not have been adequately compared. Furthermore, all centres worldwide do not follow the same neuroimaging MRI protocol which may compromise the sensitivity of MRI to detect some of the more subtle findings of OSB. Lastly, due to considerable statistical heterogeneity and clinical diversity in SR1, a direct comparison of ultrasound to MRI and meta-analysis could not be performed. This highlights the need for well-designed experiments with standardised reporting of MRI studies in OSB, to compare the two modalities in intracranial evaluation of spina bifida foetuses and neonates.

## Conclusion

OSB foetuses have a constellation of intracranial abnormalities and due to the complex nature of the disease, these anomalies can be varied with some features uncommon. This review provides systematic detail of the diagnosis by MRI of both typical (frequent) and additional intracranial findings using traditional and advanced sequences which are necessary for comprehensive pre- and post-operative assessments of a foetus with OSB. This may enable appropriate candidate selection for prenatal surgery and to compare efficacy of different prenatal surgical techniques such as laparotomy and foetoscopy. In addition, we showed that MRI has the potential to go beyond structural reporting and to provide insightful information on functional foetal brain properties that might inform our understanding of the mechanisms by which foetal surgery in OSB alters the brain. This work therefore confirms the utility of MRI as the primary imaging modality in foetal and early neonatal OSB assessment. This can assist diagnosis, prognosis, and post-operative evaluation especially when faced with suboptimal ultrasound imaging.

## Supplementary Information

Below is the link to the electronic supplementary material.

Supplementary file1 (PDF 330 KB)

Supplementary file2 (PDF 62 KB)

Supplementary file3 (PDF 76 KB)

## Declarations

### Availability of data and material

All data and material of this work are available on request from the authors.

### Code availability

Code availability is not required. A code was not used for this work because the article type is a systematic review.
