# Supplementary material for: What brain abnormalities can magnetic resonance imaging detect in foetal and early neonatal spina bifida: a systematic review
Source: Neuroradiology. 2021 Nov 18;64(2):233–45. doi: 10.1007/s00234-021-02853-1 (PMC8789702; doi:10.1007/s00234-021-02853-1)
Supplement: Supplementary file 1 — Supplementary file1 (PDF 330 KB) [file 234_2021_2853_MOESM1_ESM.pdf]

Type of Surgery	First Author and Year of Publication		Fetal Spina Bifida Patients (No.)	Fetus ≤ 26 weeks GA scans (No.)	Fetus >26 weeks GA scans (No.)	Neonatal ≤28 days scans (No.)
Postnatal Surgery	Ando 2007[30]		5	-	5	-
	Araujo 2013[2]		10	-	10	-
	Levine 2002[15]		13	9	4	-
	Saleem 2009[14]		8	3	5	-
	Walker 2016[55]		46	46 (GA not stated)		34
Fetal surgery (Open Fetal and Fetoscopic)	Vazquez 2016[56]	Open Fetal	7	7	7	7
		Fetoscopic	7	7	7	7
Both Postnatal and Open Fetal surgery	Didier 2019[31]	Postnatal	459	459 (GA not stated)		
		Open Fetal	8	8 (GA not stated)		28
	Maurice 2020[32]	Postnatal	12	12	-	-
		Open Fetal	13	13	-	-
Total Postnatal Surgery			553	24	24	34
Total Open Fetal surgery			28	20	7	35
Total Fetoscopic surgery			7	7	7	7
Total			588	51	38	76

Table 1: Included studies for systematic review 1 comparing MRI directly to ultrasound in detection of additional brain abnormalities, in patients with fetal spina bifida in the fetal and early neonatal period. GA: gestational age

Type of Surgery	First Author and Year of Publication	Fetal Spina Bifida (OSB) Patients (No.)	Closed Spina Bifida (CSB) Patients (No.)	Fetus ≤ 26 week GA scans (No.)	Fetus >26 week GA scans (No.)	Neonatal ≤28 days scans (No.)
**Postnatal Surgery**	Abele 2013[57]	37	-	-	37	-
	Appasamy 2006[58]	8	-	8 (GA not stated)		
	Batty 2012[35]	65	-	1	17	-
	Chance 2015[59]	-	1	-	-	1 (CSB)
	Hashiguchi 2015[19]	16		-		16
	Hino-Shishikura 2015[37]	66	29	-		66
	Manganaro 2012[60]	1	-	-	-	1
	Mignone Philpott 2013[47]	8	-	8	-	-
	Nagaraj 2016[52]	90	16	90	16 (CSB)	90 (OSB), 16 (CSB)
	Rangasami 2012[61]	15	-	-	15	-
	Rickard 2006[53]	17	-	17		-
	Shrot 2019[49]	26	-	26		-
	Sival 2011[62]	40	-	-	-	40
	Sweeny 2012[54]	33	-	-	-	33
	Vaikousi 2017[26]	11	-	11 (GA not stated)		
	Warner 2019[8]	20	-	20	20	-
	Woitek 2014[33]	44	13	44(OSB), 13 (CSB)	-	-
	Woitek 2016[50]	15	-	15	-	-
	Yu 2018[27]	10	-	10	-	-
	Total	**381**	-	**231 (OSB),13 (CSB)**	**89 (OSB),16 (CSB)**	**246 (OSB), 17 (CSB)**
**Open Fetal Surgery**	Bekiesinska-Figatowska 2013[63]	18	-	18	18	-
	Calle 2020[3]	29	-	29	-	-
	Mangels 2000[43]	33	-	32	1	4
	Mehollin-Ray 2014[44]	12	-	12	12	
	Nasiadko 2014[46]	9	-	9	9	

	Rethmann 2017[42]		27	-	27	27	27
	Simon 2000[64]		4	-	4		
	**Total**		**93**	-	**131**	**67**	**31**
**Fetal surgery (Fetoscopic)**	Carrabba 2019[65]		5	-	-	3	5
**Both Open Fetal and Fetoscopic Fetal Surgery**	Nagaraj 2020[45]	Open Fetal	47	-	47	5	47
		Fetoscopic	15	-	15	6	15
	Sanz-Cortes 2018[28]	Open Fetal	10	-	10	10	-
		Fetoscopic	10	-	10	10	-
	Sanz-Cortes 2020[48]	Open Fetal	30	-	16	24	-
		Fetoscopic	27	-	14	24	-
	Zarutskie 2019[51]	Open Fetal	30	-	30	30	-
		Fetoscopic	18	-	18	18	-
	**Total Open Fetal**	**Open Fetal**	**117**	-	**103**	**69**	**47**
	**Total Fetoscopic**	**Fetoscopic**	**70**	-	**57**	**58**	**15**
**Both Postnatal and Open Fetal Surgery**	Aertsen 2019[6]	Postnatal	29	-	29		-
		Open Fetal	23	-	23	23	-
	Danzer 2007[40]	Postnatal	16	-	-	16	-
		Open Fetal	22	-	22	22	-
	Grant 2011[36]	Postnatal	24	-	-	-	24
		Open Fetal	29	-	29	29	-
	Nagaraj 2017[39]	Postnatal	70	-	70	-	70
		Open Fetal	32	-	32	-	32
	Nagaraj 2016[66]	Postnatal	74	-	74	-	-
		Open Fetal	21	-	21	-	-
	**Total Postnatal Surgery**	**Postnatal**	**213**	-	**173**	**16**	**94**
	**Total Open Fetal Surgery**	**Open Fetal**	**127**	-	**127**	**74**	**32**

Fetal Spina Bifida			Fetus ≤ 26 weeks GA scans (No)	Fetus >26 weeks GA scans (No)	Neonatal ≤28 days scans (No)
**Total Postnatal Surgery**	594	-	282	118	340
**Total Open Fetal Surgery**	337	-	371	220	110
**Total Fetoscopic Fetal Surgery**	75	-	57	61	20
**Total**	**1006**	-	**710**	**399**	**470**
**Closed Spina Bifida**		-	-	-	-
**Total**	**46**	-	**13**	**16**	**17**

Table 2: Included studies for systematic review 2 on detection of brain abnormalities by MRI in fetal and early neonatal spina bifida. OSB = fetal spina bifida, CSB = closed spina bifida, GA=gestational age

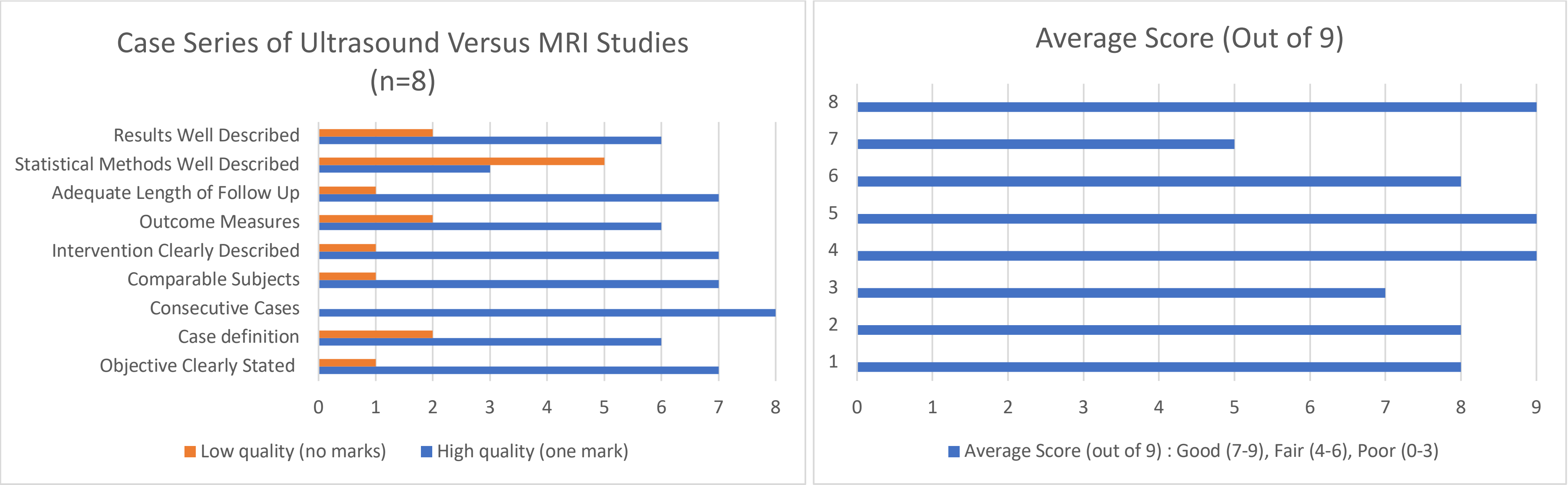

Fig. 1 Summary of risk of bias for according to study type (case-series) in the systematic review comparing MRI to ultrasound in detection of additional brain abnormalities, in fetal and early neonatal spina bifida (SR1)

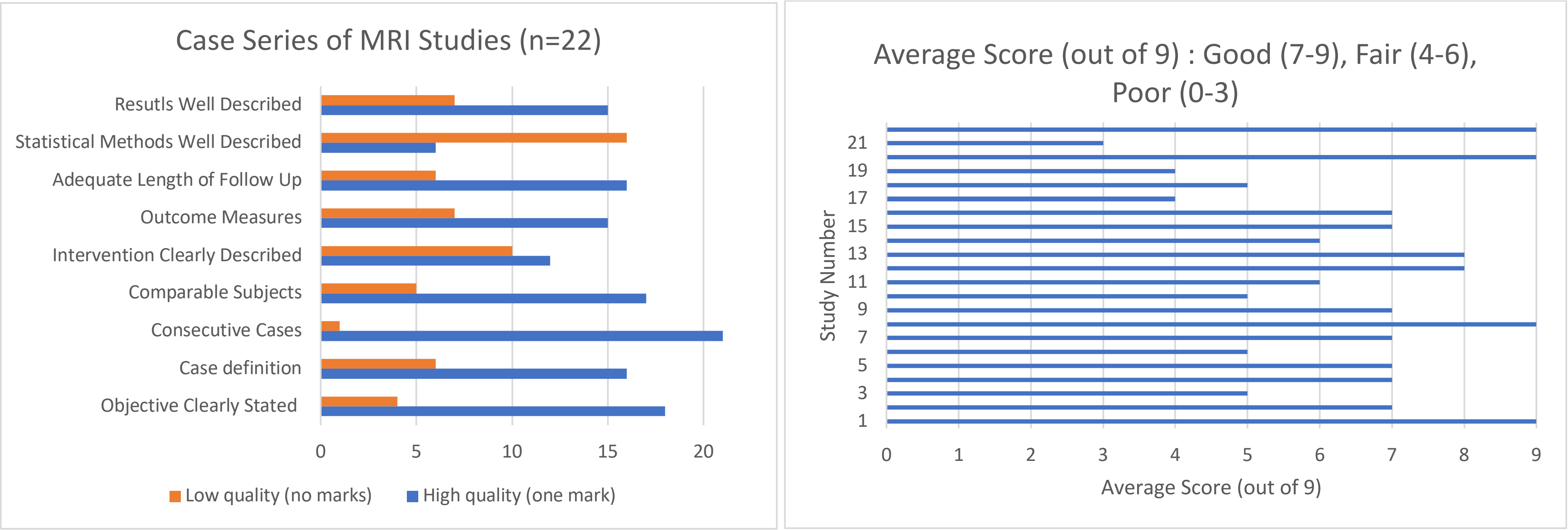

Fig. 2 Summary of risk of bias for according to study type (case-series) in the systematic review on detection of brain abnormalities by MRI in fetal and early neonatal spina bifida (SR2)

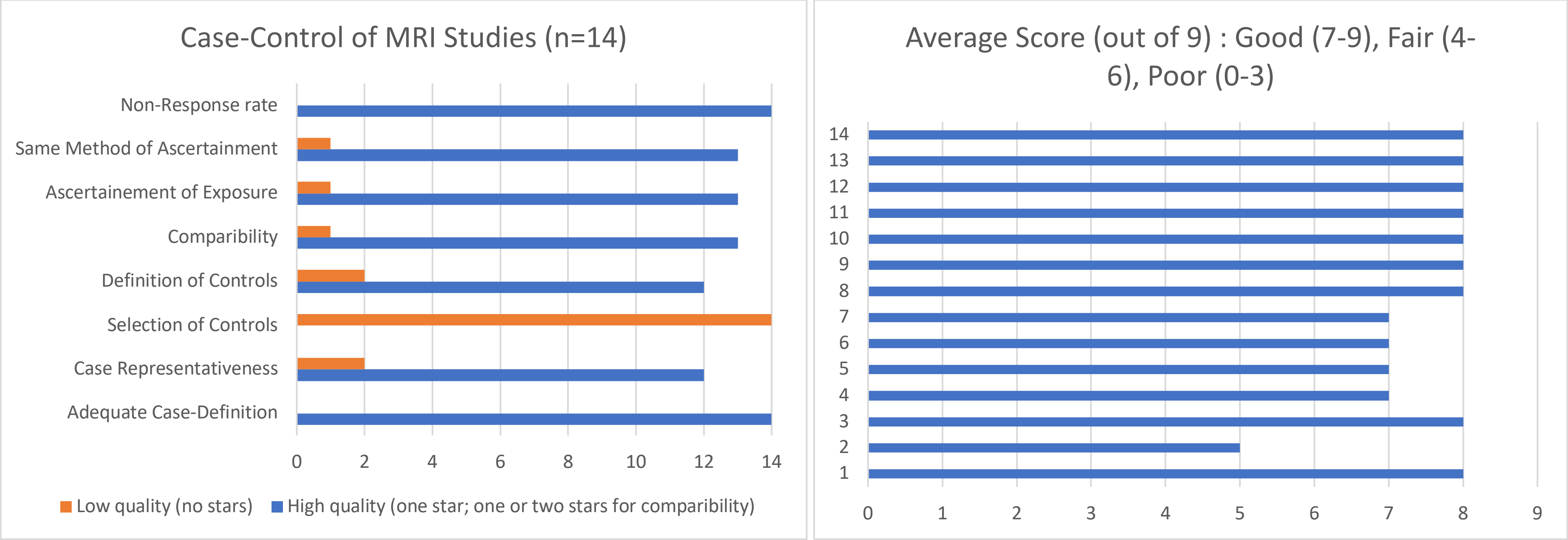

Fig. 3 Summary of risk of bias for according to study type (non-randomised study) in the systematic review on detection of brain abnormalities by MRI in fetal and early neonatal spina bifida (SR2)

Abnormality	Timepoint	Number of Studies	Event Rate	Lower Limit	Upper Limit	Heterogeneity	
						I-squared	P-Value
Callosal (Agenesis: complete/partial), Heterotopia, Hindbrain herniation, Ventriculomegaly/ Hydrocephalus	Fetus ≤26 weeks GA	22	87.5	72.5	94.9	78.875	0.000
Heterotopia, Hindbrain herniation, Ventriculomegaly/Hydrocephalus	Fetus > 26 weeks GA	9	82.2	54.5	94.7	81.908	0.000
Callosal (Agenesis: complete/partial), Heterotopia, Hindbrain herniation, Ventriculomegaly/Hydrocephalus	Neonatal ≤28 days	13	75.9	52.9	89.8	89.654	0.000
Callosal (Agenesis: complete/partial), Cerebellar Abnormalities, Hindbrain herniation, Ventriculomegaly/Hydrocephalus	All gestations	22	89.0	81.8	93.6	71.441	0.000
Callosal (Agenesis: complete/partial)	Fetus ≤26 weeks GA, Neonatal ≤ 28 days, All gestations	4	15.1	5.7	34.3	0.000	0.779
	Fetus ≤26 weeks GA	2	15.5	3.8	45.7	0.000	0.482
	Neonatal ≤ 28 days	1	25.0	3.4	76.2	0.000	1.000
	All gestations	1	9.1	1.3	43.9	0.000	1.000
Cerebellar Abnormalities	All gestations	4	88.9	22.0	99.6	85.031	0.000
Heterotopia	Fetus ≤26 weeks GA, Fetus>26 weeks GA, Neonatal ≤ 28 days	10	25.5	17.4	35.9	63.686	0.003
	Fetus ≤26 weeks GA	3	19.8	7.7	42.2	27.446	0.252
	Fetus>26 weeks GA	2	25.3	13.7	41.9	0.000	0.508
	Neonatal ≤ 28 days	5	26.9	15.3	42.8	80.157	0.000
Hindbrain herniation	All gestations	9	92.2	84.4	96.3	62.943	0.006
	Fetus ≤26 weeks GA	7	97.4	92.8	99.1	0.000	0.941
	Fetus>26 weeks GA	4	90.6	43.7	99.2	79.945	0.002
	Neonatal ≤ 28 days	3	98.9	94.7	99.8	0.000	0.940
Ventriculomegaly/Hydrocephalus	All gestations	8	87.6	77.6	93.5	50.525	0.049
	Fetus ≤26 weeks GA	10	93.2	82.5	97.5	59.447	0.008
	Fetus>26 weeks GA	3	92.5	78.8	97.6	0.000	0.378
	Neonatal ≤ 28 days	4	96.6	90.6	98.8	0.000	0.853

Table 3: Heterogeneity for all MRI Studies

**Abnormality**	**Timepoint**	**Number of Studies**	**Event Rate**	**Lower Limit**	**Upper Limit**	**Heterogeneity**	
						**I-squared**	**P-Value**
Chiari II reversal (Complete/Partial)	Fetus > 26 weeks GA, Neonatal ≤28 days, All gestations	10	85.9	66.3	94.9	87.400	0.000
	Fetus > 26 weeks GA	4	87.7	69.9	95.7	27.327	0.248
	Neonatal ≤28 days	4	92.3	60.7	99.0	72.755	0.012
	All gestations	3	76.2	22.7	97.2	93.912	0.000
Stable Ventriculomegaly Post Operatively	Fetus ≤26 weeks GA	1	11.1	1.5	50.0	0.000	1.000
Reduced Ventriculomegaly Post Operatively	Fetus > 26 weeks GA	2	6.3	1.6	22.2	0.000	0.423
Increased Ventriculomegaly Post Operatively	Fetus >26 weeks GA, Neonatal ≤28 days	6	71.7	44.6	88.8	76.171	0.001
	Fetus >26 weeks GA	5	76.8	63.1	86.4	0.000	0.448
	Neonatal ≤28 days	2	43.4	23.8	65.3	70.968	0.063

Table 4: Heterogeneity for Post-Operative Findings

**Characteristics**			**Statistics for each study**			
**Time Point**	**Abnormality**	**Study Name (Author, year, Type of Surgery)**	**Event/Total**	**Event rate (%)**	**Lower Limit (%)**	**Upper Limit (%)**
Fetus ≤ 26 Weeks GA	Callosal Abnormalities (Agenesis - Complete or Partial)	Yu 2018 (Postnatal Surgery)[27]	1 / 10	10.0	1.4	46.7
		Simon 2000 (Open Fetal Surgery)[16]	1 / 4	25.0	3.4	76.2
	Heterotopia	Nasiadko 2014 (Open Fetal Surgery)[25]	1 / 9	11.1	1.5	50.0
		Rethmann 2017 (Open Fetal Surgery)[42]	4 / 27	14.8	5.7	33.5
		Simon 2000 (Open Fetal Surgery)[16]	2 / 4	50.0	12.3	87.7
	Hindbrain Herniation	Yu 2018 (Postnatal Surgery)[27]	10 / 10	95.5	55.2	99.7
		Calle 2020 (Open Fetal Surgery)[3]	29 / 29	98.3	78.3	99.9
		Simon 2000 (Open Fetal Surgery)[16]	4 / 4	90.0	32.6	99.4
		Nagaraj 2020 (Open Fetal Surgery)[45]	47 / 47	99.0	85.4	99.9
		Nagaraj 2020 (Fetoscopic Fetal Surgery)[45]	15 / 15	96.9	65.0	99.8

		Aertsen 2019 (Open Fetal Surgery)[6]	23 / 23	97.9	74.1	99.9
		Aertsen 2019 (Postnatal Surgery)[6]	29 / 29	98.3	78.3	99.9
	Ventriculomegaly/Hydrocephalus	Batty 2012 (Postnatal Surgery)[35]	48 / 48	99.0	85.7	99.9
		Rickard 2006 (Postnatal Surgery)[53]	14 / 17	82.4	57.3	94.2
		Shrot 2006 (Postnatal Surgery)[49]	16 / 26	61.5	42.1	77.9
		Yu 2018 (Postnatal Surgery)[27]	10 / 10	95.5	55.2	99.7
		Nasiadko 2014 (Open Fetal Surgery)[25]	9 / 9	95.0	52.5	99.7
		Rethmann 2017 (Open Fetal Surgery)[42]	27 / 27	98.2	77.0	99.9
		Sanz-Cortes 2018 (Open Fetal Surgery)[28]	10 / 10	95.5	55.2	99.7
		Sanz-Cortes 2018 (Fetoscopic Fetal Surgery)[28]	10 / 10	95.5	55.2	99.7
		Sanz-Cortes 2020 (Open Fetal Surgery)[48]	16 / 16	97.1	66.4	99.8
		Sanz-Cortes 2020 (Fetoscopic Fetal Surgery)[48]	14 / 14	96.7	63.4	99.8
**Cumulative Study Statistics**			**327/398**	**87.5**	**72.5**	**94.9**
**Characteristics**			**Statistics for each study**			
**Time Point**	**Abnormality**	**Study Name (Author, year, Type of Surgery)**	**Event/Total**	**Event rate (%)**	**Lower Limit (%)**	**Upper Limit (%)**
Fetus > 26 Weeks GA	Heterotopia	Nasiadko 2014 (Open Fetal Surgery)[25]	3 / 9	33.3	11.1	66.7
		Rethmann 2017 (Open Fetal Surgery)[42]	6 / 27	22.2	10.3	41.4
	Hindbrain Herniation	Abele 2013 (Postnatal Surgery)[57]	37 / 37	98.7	82.2	99.9
		Carrabba 2019 (Fetoscopic Fetal Surgery)[65]	5 / 5	91.7	37.8	99.5
		Hashiguchi 2015 (Postnatal Surgery)[65]	7 / 16	43.8	22.5	67.6
		Rangasami 2012 (Postnatal Surgery)[61]	15 / 15	96.9	65.0	99.8
	Ventriculomegaly/Hydrocephalus	Warner 2019 (Postnatal Surgery)[8]	20 / 20	97.6	71.3	99.9
		Batty 2012 (Postnatal Surgery)[35]	17 / 17	97.2	67.8	99.8
		Rangasami 2012 (Postnatal Surgery)[61]	13 / 15	86.7	59.5	96.6
**Cumulative Study Statistics**			**123/161**	**82.2**	**54.5**	**94.7**
**Characteristics**			**Statistics for each study**			
**Time Point**	**Abnormality**	**Study Name (Author, year, Type of Surgery)**	**Event/Total**	**Event rate (%)**	**Lower Limit (%)**	**Upper Limit (%)**
Neonatal ≤28 days	Callosal Abnormalities (Agenesis - Complete or Partial)	Mangels 2000 (Open Fetal Surgery)[17]	1 / 4	25.0	3.4	76.2
	Heterotopia	Rethmann 2017 (Open Fetal Surgery)[42]	12 / 27	44.4	27.2	63.1
		Nagaraj 2020 (Open Fetal Surgery)[45]	19 / 47	40.4	27.5	54.9
		Nagaraj 2020 (Fetoscopic Fetal Surgery)[45]	2 / 15	13.3	3.4	40.5
		Nagaraj 2016 (Open Fetal Surgery and Postnatal Surgery)[66]	11 / 95	11.6	6.5	19.7
		Hino-Shishijura 2015 (Postnatal Surgery)[37]	20 / 66	30.3	20.5	42.4

	Hindbrain Herniation	Sival 2011 (Postnatal Surgery)[62]	40 / 40	98.8	83.3	99.9
		Hino-Shishijura 2015 (Postnatal Surgery)[37]	66 / 66	99.3	89.2	100.0
		Sweeny 2012 (Postnatal Surgery)[54]	33 / 33	98.5	80.4	99.9
	Ventriculomegaly/Hydrocephalus	Danzer 2007 (Open Fetal Surgery)[40]	22 / 22	97.8	73.2	99.9
		Danzer 2007 (Postnatal Surgery)[40]	16 / 16	97.1	66.4	99.8
		Sival 2011 (Postnatal Surgery)[62]	38 / 40	95.0	82.1	98.7
		Sweeny 2012 (Postnatal Surgery)[54]	33 / 33	98.5	80.4	99.9
**Cumulative Study Statistics**			**313/504**	**75.9**	**52.9**	**89.8**
**Characteristics**			**Statistics for each study**			
**Time Point**	**Abnormality**	**Study Name (Author, year, Type of Surgery)**	**Event/Total**	**Event rate (%)**	**Lower Limit (%)**	**Upper Limit (%)**
All gestations		Vaikousi 2017 (Postnatal Surgery)[26]	1 / 11	9.1	1.3	43.9
	Cerebellar Abnormalities	Danzer 2007 (Open Fetal Surgery)[40]	22 / 22	97.8	73.2	99.9
		Danzer 2007 (Postnatal Surgery)[40]	16 / 16	97.1	66.4	99.8
		Vaikousi 2017 (Postnatal Surgery)[26]	1 / 11	9.1	1.3	43.9
		Warner 2019 (Postnatal Surgery)[8]	20 / 20	97.6	71.3	99.9
	Hindbrain Herniation	Batty 2012 (Postnatal Surgery)[35]	50 / 65	6.9	65.2	85.6
		Mangels 2000 (Open Fetal Surgery)[17]	37 / 37	98.7	82.2	99.9
		Grant 2011 (Open Fetal Surgery)[36]	29 / 29	98.3	78.3	99.9
		Nagaraj 2016 (Open Fetal Surgery and Postnatal Surgery)[66]	87 / 95	91.6	84.1	95.7
		Nagaraj 2016 (Postnatal Surgery)[52]	86 / 90	95.6	88.8	98.3
		Mehollin-Ray 2014 (Open Fetal Surgery)[44]	11 / 12	91.7	58.7	98.8
		Warner 2019 (Postnatal Surgery)[8]	20 / 20	97.6	71.3	99.9
		Bekiesinska-Figatowska 2013 (Open Fetal Surgery)[63]	18 / 18	97.4	69.0	99.8
		Appasamy 2006 (Postnatal Surgery)[58]	6 / 8	75.0	37.7	93.7
	Ventriculomegaly/Hydrocephalus	Mangels 2000 (Open Fetal Surgery)[17]	37 / 37	98.7	82.2	99.9
		Grant 2011 (Postnatal Surgery)[36]	24 / 24	98.0	74.9	99.9
		Nagaraj 2016 (Open Fetal Surgery and Postnatal Surgery)[66]	75 / 95	78.9	69.6	86.0
		Hashiguchi 2015 (Postnatal Surgery)[19]	16 / 16	97.1	66.4	99.8
		Nagaraj 2016 (Postnatal Surgery)[52]	77 / 90	85.6	76.7	91.4
		Mehollin-Ray 2014 (Open Fetal Surgery)[44]	12 / 12	96.2	59.7	99.8
		Vaikousi 2017 (Postnatal Surgery)[26]	11 / 11	95.8	57.5	99.7
		Appasamy 2006 (Postnatal Surgery)[58]	5 / 8	62.5	28.5	87.5
**Cumulative Study Statistics**			**661/747**	**89.0**	**81.8**	**93.6**

Characteristics			Statistics for each study			
**Time Point**	**Abnormality**	**Study Name (Author, year, Type of Surgery)**	**Event/Total**	**Event rate (%)**	**Lower Limit (%)**	**Upper Limit (%)**
Fetus ≤26 weeks, Neonatal ≤ 28 days, All gestations	Callosal Abnormalities (Agenesis - Complete or Partial)	Vaikousi 2017 (Postnatal Surgery)[26]	1 / 11	9.1	1.3	43.9
		Yu 2018 (Postnatal Surgery)[27]	1 / 10	10.0	1.4	46.7
		Simon 2000 (Open Fetal Surgery)[16]	1 / 4	25.0	3.4	76.2
		Mangels 2000 (Open Fetal Surgery)[17]	1 / 4	25.0	3.4	76.2
**Cumulative Study Statistics**			**4/29**	**15.1**	**5.7**	**34.3**
**Characteristics**			**Statistics for each study**			
**Time Point**	**Abnormality**	**Study Name (Author, year, Type of Surgery)**	**Event/Total**	**Event rate (%)**	**Lower Limit (%)**	**Upper Limit (%)**
Fetus ≤26 weeks GA	Callosal Abnormalities (Agenesis - Complete or Partial)	Simon 2000 (Open Fetal Surgery)[16]	1 / 4	25.0	3.4	76.2
		Yu 2018 (Postnatal Surgery)[27]	1 / 10	10.0	1.4	46.7
**Cumulative Study Statistics**			**2/14**	**15.5**	**3.8**	**45.7**
**Characteristics**			**Statistics for each study**			
**Time Point**	**Abnormality**	**Study Name (Author, year, Type of Surgery)**	**Event/Total**	**Event rate (%)**	**Lower Limit (%)**	**Upper Limit (%)**
Neonatal ≤28 days	Callosal Abnormalities (Agenesis - Complete or Partial)	Mangels 2000 (Open Fetal Surgery)[17]	**1/4**	25.0	3.4	76.2
**Cumulative Study Statistics †a**			**1/4**	**25.0**	**3.4**	**76.2**
**Characteristics**			**Statistics for each study**			
**Time Point**	**Abnormality**	**Study Name (Author, year, Type of Surgery)**	**Event/Total**	**Event rate (%)**	**Lower Limit (%)**	**Upper Limit (%)**
All gestations	Callosal Abnormalities (Agenesis - Complete or Partial)	Vaikousi 2017 (Postnatal Surgery)[26]	1/11	9.1	1.3	43.9
**Cumulative Study Statistics †a**			**1/11**	**9.1**	**1.3**	**43.9**
**Characteristics**			**Statistics for each study**			
**Time Point**	**Abnormality**	**Study Name (Author, year, Type of Surgery)**	**Event/Total**	**Event rate (%)**	**Lower Limit (%)**	**Upper Limit (%)**
All gestations	Cerebellar Abnormalities	Danzer 2007 (Open Fetal Surgery)[40]	22 / 22	97.8	73.2	99.9
		Danzer 2007 (Postnatal Surgery)[40]	16 / 16	97.1	66.4	99.8
		Vaikousi 2017 (Postnatal Surgery)[26]	1 / 11	9.1	1.3	43.9
		Warner 2019 (Postnatal Surgery)[8]	20 / 20	97.6	71.3	99.9
Cumulative Study Statistics			**59/69**	**88.9**	**22.0**	**99.6**

Characteristics			Statistics for each study			
**Time Point**	**Abnormality**	**Study Name (Author, year, Type of Surgery)**	**Event/Total**	**Event rate (%)**	**Lower Limit (%)**	**Upper Limit (%)**
Fetus ≤26 weeks GA, Fetus>26 weeks GA, Neonatal ≤ 28 days	Heterotopia	Nasiadko 2014 (Open Fetal Surgery)[25]	1 / 9	11.1	1.5	50.0
		Rethmann 2017 (Open Fetal Surgery)[42]	4 / 27	14.8	5.7	33.5
		Simon 2000 (Open Fetal Surgery)[16]	2 / 4	50.0	12.3	87.7
		Nasiadko 2014 (Open Fetal Surgery)[25]	3 / 9	33.3	11.1	66.7
		Rethmann 2017 (Open Fetal Surgery)[42]	6 / 27	22.2	10.3	41.4
		Hino-Shishijura 2015 (Postnatal Surgery)[37]	20 / 66	30.3	20.5	42.4
		Nagaraj 2016 (Open Fetal Surgery and Postnatal Surgery)[66]	11 / 95	11.6	6.5	19.7
		Nagaraj 2020 (Fetoscopic Fetal Surgery)[45]	2 / 15	13.3	3.4	40.5
		Nagaraj 2020 (Open Fetal Surgery)[45]	19 / 47	40.4	27.5	54.9
		Rethmann 2017 (Open Fetal Surgery)[42]	12 / 27	44.4	27.2	63.1
**Cumulative Study Statistics**			**80/326**	**25.5**	**17.4**	**35.9**
**Characteristics**			**Statistics for each study**			
**Time Point**	**Abnormality**	**Study Name (Author, year, Type of Surgery)**	**Event/Total**	**Event rate (%)**	**Lower Limit (%)**	**Upper Limit (%)**
Fetus ≤26 weeks GA	Heterotopia	Nasiadko 2014 (Open Fetal Surgery)[25]	1 / 9	11.1	1.5	50.0
		Rethmann 2017 (Open Fetal Surgery)[42]	4 / 27	14.8	5.7	33.5
		Simon 2000 (Open Fetal Surgery)[16]	2 / 4	50.0	12.3	87.7
**Cumulative Study Statistics**			**7/40**	**19.8**	**7.7**	**42.2**
**Characteristics**			**Statistics for each study**			
**Time Point**	**Abnormality**	**Study Name (Author, year, Type of Surgery)**	**Event/Total**	**Event rate (%)**	**Lower Limit (%)**	**Upper Limit (%)**
Fetus>26 weeks GA	Heterotopia	Nasiadko 2014 (Open Fetal Surgery)[25]	3 / 9	33.3	11.1	66.7
		Rethmann 2017 (Open Fetal Surgery)[42]	6 / 27	22.2	10.3	41.4
**Cumulative Study Statistics**			**9/36**	**25.3**	**13.7**	**41.9**
**Characteristics**			**Statistics for each study**			
**Time Point**	**Abnormality**	**Study Name (Author, year, Type of Surgery)**	**Event/Total**	**Event rate (%)**	**Lower Limit (%)**	**Upper Limit (%)**
Neonatal ≤ 28 days	Heterotopia	Hino-Shishijura 2015 (Postnatal Surgery)[37]	20 / 66	30.3	20.5	42.4
		Nagaraj 2016 (Open Fetal Surgery and Postnatal Surgery)[66]	11 / 95	11.6	6.5	19.7
		Nagaraj 2020 (Fetoscopic Fetal Surgery)[45]	2 / 15	13.3	3.4	40.5
		Nagaraj 2020 (Open Fetal Surgery)[45]	19 / 47	40.4	27.5	54.9
		Rethmann 2017 (Open Fetal Surgery)[42]	12 / 27	44.4	27.2	63.1

Cumulative Study Statistics			64/250	26.9	15.3	42.8
**Characteristics**			**Statistics for each study**			
**Time Point**	**Abnormality**	**Study Name (Author, year, Type of Surgery)**	**Event/Total**	**Event rate (%)**	**Lower Limit (%)**	**Upper Limit (%)**
All gestations	Hindbrain Herniation	Appasamy 2006 (Postnatal Surgery)[58]	6 / 8	75.0	37.7	93.7
		Batty 2012 (Postnatal Surgery)[35]	50 / 65	76.9	65.2	85.6
		Bekiesinska-Figatowska 2013 (Open Fetal Surgery)[63]	18 / 18	97.4	69.0	99.8
		Grant 2011 (Open Fetal Surgery)[36]	29 / 29	98.3	78.3	99.9
		Mangels 2000 (Open Fetal Surgery)[17]	37 / 37	98.7	82.2	99.9
		Mehollin-Ray 2014 (Open Fetal Surgery)[44]	11 / 12	91.7	58.7	98.8
		Nagaraj 2016 (Open Fetal Surgery and Postnatal Surgery)[66]	87 / 95	91.6	84.1	95.7
		Nagaraj 2016 (Postnatal Surgery)[52]	86 / 90	95.6	88.8	98.3
		Warner 2019 (Postnatal Surgery)[8]	20 / 20	97.6	71.3	99.9
**Cumulative Study Statistics**			**344/374**	**92.2**	**84.4**	**96.3**
**Characteristics**			**Statistics for each study**			
**Time Point**	**Abnormality**	**Study Name (Author, year, Type of Surgery)**	**Event/Total**	**Event rate (%)**	**Lower Limit (%)**	**Upper Limit (%)**
Fetus ≤26 weeks GA	Hindbrain Herniation	Aertsen 2019 (Open Fetal Surgery)[6]	23 / 23	97.9	74.1	99.9
		Aertsen 2019 (Postnatal Surgery)[6]	29 / 29	98.3	78.3	99.9
		Calle 2020 (Open Fetal Surgery)[3]	29 / 29	98.3	78.3	99.9
		Nagaraj 2020 (Fetoscopic Fetal Surgery)[45]	15 / 15	96.9	65.0	99.8
		Nagaraj 2020 (Open Fetal Surgery)[45]	47 / 47	99.0	85.4	99.9
		Simon 2000 (Open Fetal Surgery)[16]	4 / 4	90.0	32.6	99.4
		Yu 2018 (Postnatal Surgery)[27]	10 / 10	95.5	55.2	99.7
**Cumulative Study Statistics**			**157/157**	**97.4**	**92.8**	**99.1**
**Characteristics**			**Statistics for each study**			
**Time Point**	**Abnormality**	**Study Name (Author, year, Type of Surgery)**	**Event/Total**	**Event rate (%)**	**Lower Limit (%)**	**Upper Limit (%)**
Fetus>26 weeks GA	Hindbrain Herniation	Abele 2013 (Postnatal Surgery)[57]	37 / 37	98.7	82.2	99.9
		Carrabba 2019 (Fetoscopic Fetal Surgery)[65]	5 / 5	91.7	37.8	99.5
		Hashiguchi 2015 (Postnatal Surgery)[19]	7 / 16	43.8	22.5	67.6
		Rangasami 2012 (Postnatal Surgery)[61]	15 / 15	96.9	65.0	99.8
**Cumulative Study Statistics**			**64/73**	**90.6**	**43.7**	**99.2**

Characteristics			Statistics for each study			
**Time Point**	**Abnormality**	**Study Name (Author, year, Type of Surgery)**	**Event/Total**	**Event rate (%)**	**Lower Limit (%)**	**Upper Limit (%)**
Neonatal ≤ 28 days	Hindbrain Herniation	Hino-Shishijura 2015 (Postnatal Surgery)[37]	66 / 66	99.3	89.2	100.0
		Sival 2011 (Postnatal Surgery)[62]	40 / 40	98.8	83.3	99.9
		Sweeny 2012 (Postnatal Surgery)[54]	33 / 33	98.5	80.4	99.9
Cumulative Study Statistics			**139/139**	**98.9**	**94.7**	**99.8**
Characteristics			**Statistics for each study**			
Time Point	**Abnormality**	**Study Name (Author, year, Type of Surgery)**	**Event/Total**	**Event rate (%)**	**Lower Limit (%)**	**Upper Limit (%)**
All gestations	Ventriculomegaly/Hydrocephalus	Appasamy 2006 (Postnatal Surgery)[58]	5 / 8	62.5	28.5	87.5
		Grant 2011 (Postnatal Surgery)[36]	24 / 24	98.0	74.9	99.9
		Hashiguchi 2015 (Postnatal Surgery)[19]	16 / 16	97.1	66.4	99.8
		Mangels 2000 (Open Fetal Surgery)[17]	37 / 37	98.7	82.2	99.9
		Mehollin-Ray 2014 (Open Fetal Surgery)[44]	12 / 12	96.2	59.7	99.8
		Nagaraj 2016 (Open Fetal Surgery and Postnatal Surgery)[66]	75 / 95	78.9	69.6	86.0
		Nagaraj 2016 (Postnatal Surgery)[52]	77 / 90	85.6	76.7	91.4
		Vaikousi 2017 (Postnatal Surgery)[26]	11 / 11	95.8	57.5	99.7
**Cumulative Study Statistics**			**257/293**	**87.6**	**77.6**	**93.5**
**Characteristics**			**Statistics for each study**			
**Time Point**	**Abnormality**	**Study Name (Author, year, Type of Surgery)**	**Event/Total**	**Event rate (%)**	**Lower Limit (%)**	**Upper Limit (%)**
Fetus ≤26 weeks GA	Ventriculomegaly/Hydrocephalus	Batty 2012 (Postnatal Surgery)[35]	48 / 48	99.0	85.7	99.9
		Nasiadko 2014 (Open Fetal Surgery)[25]	9 / 9	95.0	52.5	99.7
		Rethmann 2017 (Open Fetal Surgery)[42]	27 / 27	98.2	77.0	99.9
		Rickard 2006 (Postnatal Surgery)[53]	14 / 17	82.4	57.3	94.2
		Sanz-Cortes 2018 (Fetoscopic Fetal Surgery)[28]	10 / 10	95.5	55.2	99.7
		Sanz-Cortes 2018 (Open Fetal Surgery)[28]	10 / 10	95.5	55.2	99.7
		Sanz-Cortes 2020 (Fetoscopic Fetal Surgery)[48]	14 / 14	96.7	63.4	99.8
		Sanz-Cortes 2020 (Open Fetal Surgery)[48]	16 / 16	97.1	66.4	99.8
		Shrot 2006 (Postnatal Surgery)[49]	16 / 26	61.5	42.1	77.9
		Yu 2018 (Postnatal Surgery)[27]	10 / 10	95.5	55.2	99.7
**Cumulative Study Statistics**			**174/196**	**93.2**	**82.5**	**97.5**
**Characteristics**			**Statistics for each study**			

Time Point	Abnormality	Study Name (Author, year, Type of Surgery)	Event/Total	Event rate (%)	Lower Limit (%)	Upper Limit (%)
Fetus>26 weeks GA	Ventriculomegaly/Hydrocephalus	Batty 2012 (Postnatal Surgery)[35]	17 / 17	97.2	67.8	99.8
		Rangasami 2012 (Postnatal Surgery)[61]	13 / 15	86.7	59.5	96.6
		Warner 2019 (Postnatal Surgery)[8]	20 / 20	97.6	71.3	99.9
**Cumulative Study Statistics**			**50/52**	**92.5**	**78.8**	**97.6**
**Characteristics**			**Statistics for each study**			
**Time Point**	**Abnormality**	**Study Name (Author, year, Type of Surgery)**	**Event/Total**	**Event rate (%)**	**Lower Limit (%)**	**Upper Limit (%)**
Neonatal ≤ 28 days	Ventriculomegaly/Hydrocephalus	Danzer 2007 (Open Fetal Surgery)[40]	22 / 22	97.8	73.2	99.9
		Danzer 2007 (Postnatal Surgery)[40]	16 / 16	97.1	66.4	99.8
		Sival 2011 (Postnatal Surgery)[62]	38 / 40	95.0	82.1	98.7
		Sweeny 2012 (Postnatal Surgery)[54]	33 / 33	98.5	80.4	99.9
**Cumulative Study Statistics**			**109/111**	**96.6**	**90.6**	**98.8**

Table 5: Meta-Analysis for all MRI studies.
†a Meta-Analysis not possible as reported by a single study.

**Characteristics**			**Statistics for each study**			
**Time Point**	**Abnormality**	**Study Name (Author, year, Type of Surgery)**	**Event/Total**	**Event rate (%)**	**Lower Limit (%)**	**Upper Limit (%)**
**Fetus >26 weeks GA, Neonatal ≤28 days, All gestations**	Chiari II reversal (Complete/Partial)	Mehollin-Ray 2014 (Open Fetal Surgery)[44]	11 / 12	91.7	58.7	98.8
		Nasiadko 2014 (Open Fetal Surgery)[25]	9 / 9	95.0	52.5	99.7
		Rethmann 2017 (Open Fetal Surgery)[42]	25 / 27	92.6	74.8	98.1
		Carrabba 2019 (Fetoscopic Fetal Surgery)[65]	3 / 5	60.0	20.0	90.0
		Nagaraj 2020 (Open Fetal Surgery)[45]	47 / 47	99.0	85.4	99.9
		Nagaraj 2020 (Fetoscopic Fetal Surgery)[45]	15 / 15	96.9	65.0	99.8
		Zarutskie 2019 (Open and Fetoscopic Fetal Surgery)[51]	32 / 48	66.7	52.3	78.5
		Grant 2011 (Open Fetal Surgery)[36]	29 / 29	98.3	78.3	99.9
		Nagaraj 2017 (Open Fetal Surgery)[39]	26 / 32	81.3	64.1	91.3
		Nagaraj 2017 (Postnatal Surgery)[39]	19 / 74	25.7	17.0	36.8

**Cumulative Study Statistics**			**216/298**	**85.9**	**66.3**	**94.9**
**Characteristics**			**Statistics for each study**			
**Time Point**	**Abnormality**	**Study Name (Author, year, Type of Surgery)**	**Event/Total**	**Event rate (%)**	**Lower Limit (%)**	**Upper Limit (%)**
**Fetus > 26 weeks GA**	Chiari II reversal (Complete/Partial)	Carrabba 2019 (Fetoscopic Fetal Surgery)[65]	3 / 5	60.0	20.0	90.0
		Mehollin-Ray 2014 (Open Fetal Surgery)[44]	11 / 12	91.7	58.7	98.8
		Nasiadko 2014 (Open Fetal Surgery)[25]	9 / 9	95.0	52.5	99.7
		Rethmann 2017 (Open Fetal Surgery)[42]	25 / 27	92.6	74.8	98.1
**Cumulative Study Statistics**			**48/50**	**87.7**	**69.9**	**95.7**
**Characteristics**			**Statistics for each study**			
**Time Point**	**Abnormality**	**Study Name (Author, year, Type of Surgery)**	**Event/Total**	**Event rate (%)**	**Lower Limit (%)**	**Upper Limit (%)**
**Neonatal ≤ 28 days**	Chiari II reversal (Complete/Partial)	Carrabba 2019 (Fetoscopic Fetal Surgery)[65]	5 / 5	91.7	37.8	99.5
		Nagaraj 2020 (Fetoscopic Fetal Surgery)[45]	15 / 15	96.9	65.0	99.8
		Nagaraj 2020 (Open Fetal Surgery)[45]	47 / 47	99.0	85.4	99.9
		Zarutskie 2019 (Open and Fetoscopic Fetal Surgery)[51]	32 / 48	66.7	52.3	78.5
**Cumulative Study Statistics**			**99/115**	**92.3**	**60.7**	**99.0**
**Characteristics**			**Statistics for each study**			
**Time Point**	**Abnormality**	**Study Name (Author, year, Type of Surgery)**	**Event/Total**	**Event rate (%)**	**Lower Limit (%)**	**Upper Limit (%)**
**All gestations**	Chiari II reversal (Complete/Partial**)**	Grant 2011 (Open Fetal Surgery)[36]	29 / 29	98.3	78.3	99.9
		Nagaraj 2017 (Open Fetal Surgery)[39]	26 / 32	81.3	64.1	91.3
		Nagaraj 2017 (Postnatal Surgery)[39]	19 / 74	25.7	17.0	36.8
**Cumulative Study Statistics**			**74/135**	**76.2**	**22.7**	**97.2**
**Characteristics**			**Statistics for each study**			

Time Point	Abnormality	Study Name (Author, year, Type of Surgery)	Event/Total	Event rate (%)	Lower Limit (%)	Upper Limit (%)
**Fetus >26 weeks GA**	Stable Ventriculomegaly Post Operatively	Nasiadko 2014 (Open Fetal Surgery)[25]	**1 / 9**	11.1	1.5	50.0
**Cumulative Study Statistics †a**			**1 / 9**	**11.1**	**1.5**	**50.0**
**Characteristics**			**Statistics for each study**			
**Time Point**	**Abnormality**	**Study Name (Author, year, Type of Surgery)**	**Event/Total**	**Event rate (%)**	**Lower Limit (%)**	**Upper Limit (%)**
**Fetus > 26 weeks GA**	Reduced Ventriculomegaly Post Operatively	Nasiadko 2014 (Open Fetal Surgery)[25]	1 / 9	11.1	1.5	50.0
		Rethmann 2017 (Open Fetal Surgery)[42]	1 / 27	3.7	0.5	22.1
**Cumulative Study Statistics**			**2/36**	**6.3**	**1.6**	**22.2**
**Characteristics**			**Statistics for each study**			
**Time Point**	**Abnormality**	**Study Name (Author, year, Type of Surgery)**	**Event/Total**	**Event rate (%)**	**Lower Limit (%)**	**Upper Limit (%)**
**Fetus >26 weeks GA, Neonatal ≤28 days**	Increased Ventriculomegaly Post Operatively	Mehollin-Ray 2014 (Open Fetal Surgery)[44]	12 / 12	96.2	59.7	99.8
		Nagaraj 2020 (Fetoscopic Fetal Surgery)[45]	6 / 6	92.9	42.3	99.6
		Nagaraj 2020 (Open Fetal Surgery)[45]	3 / 5	60.0	20.0	90.0
		Nasiadko 2014 (Open Fetal Surgery)[25]	7 / 9	77.8	42.1	94.4
		Rethmann 2017 (Open Fetal Surgery)[42]	20 / 27	74.1	54.7	87.1
		Zarutskie 2019 (Open and Fetoscopic Fetal Surgery)[51]	16 / 48	33.3	21.5	47.7
**Cumulative Study Statistics**			**64/107**	**71.7**	**44.6**	**88.8**
**Characteristics**			**Statistics for each study**			
**Time Point**	**Abnormality**	**Study Name (Author, year, Type of Surgery)**	**Event/Total**	**Event rate (%)**	**Lower Limit (%)**	**Upper Limit (%)**
**Fetus >26 weeks GA**	Increased Ventriculomegaly Post Operatively	Mehollin-Ray 2014 (Open Fetal Surgery)[44]	12 / 12	96.2	59.7	99.8
		Nagaraj 2020 (Fetoscopic Fetal Surgery)[45]	6 / 6	92.9	42.3	99.6
		Nagaraj 2020 (Open Fetal Surgery)[45]	3 / 5	60.0	20.0	90.0

		Nasiadko 2014 (Open Fetal Surgery)[25]	7 / 9	77.8	42.1	94.4
		Rethmann 2017 (Open Fetal Surgery)[42]	20 / 27	74.1	54.7	87.1
**Cumulative Study Statistics**			**48/59**	**76.8**	**63.1**	**86.4**
**Characteristics**			**Statistics for each study**			
**Time Point**	**Abnormality**	**Study Name (Author, year, Type of Surgery)**	**Event/Total**	**Event rate (%)**	**Lower Limit (%)**	**Upper Limit (%)**
**Neonatal ≤28 days**	Increased Ventriculomegaly Post Operatively	Rethmann 2017 (Open Fetal Surgery)[42]	15 / 27	55.6	36.9	72.8
		Zarutskie 2019 (Open and Fetoscopic Fetal Surgery)[51]	16 / 48	33.3	21.5	47.7
Cumulative Study Statistics			**31/75**	**43.4**	**23.8**	**65.3**

Table 6: Meta-Analysis for Intracranial Post-operative findings
†a Meta-Analysis not possible as reported by a single study

**Reported Abnormality**	**First Author, Year of Publication and timing of MMC surgery**	**Description n/N (%) or *p* value**
Posterior Fossae Measurements	Woitek 2014 (Postnatal Surgery[33]	• Clivus supra-occiput angle (CSA) and transverse diameter of the posterior fossae (TDPF) of MMC was significantly smaller than controls (p≤0.001)<br>• TDPF in closed spina bifida was significantly smaller in closed spina bifida patients than in controls (p≤0.001)
	Aertsen 2019 (Postnatal and Fetal Surgery)[6]	• CSO, TDPF, Transverse cerebellar diameter (TCD), and posterior fossae area (PFA) was significantly different between MMC (who were operated on postnatally), and MMC (who had open Fetal surgery) versus controls (p≤0.0001)
	Batty 2012 (Postnatal Surgery)[35]	• PFA was reduced in MMC patients
	Warner 2019 (Postnatal Surgery)[8]	• Bony PFA was smaller in MMC patients, at 20-24 weeks (p≤0.001), and 30-32 weeks (p≤0.001)

	Grant 2011 (Postnatal Surgery)[36]	• CSA was acute in MMC patients but was comparable to controls at birth 24/70 (34.3%)
Anatomical Descriptive markers of Chiari II malformation	Hino-Shishikura 2015 (Postnatal Surgery)[37]	• Lower level of medullary kink and low pontomesencephalic junction in MMC patients with cortical malformations 20/66 (30.3%)
	Calle 2020 (Fetal Open Surgery)[3]	• Downward herniation of the cerebellum 29/29 (100%)<br>• Towering cerebellum 6/29 (20.7%)<br>• Intermediate towering of the cerebellum 9/29 (31%)<br>• Downward herniation of the brainstem 18/29 (62.1%)<br>• Intermediate herniation of the brainstem 9/29 (31%)<br>• Flattening or deformity of the fourth ventricle 28/29 (96.6%)<br>• Tectal beaking 7/29 (24.1%)<br>• Intermediate tectal beaking 20/29 (69%)<br>• Hypoplastic tentorium 29/29 (100%)

Table 7: Additional infratentorial intracranial findings using traditional MRI sequences.

**Reported Abnormality**	**First Author, Year of Publication and timing of MMC surgery**	**Description n/N (%) or *p* value**
Ventricular Abnormality	Rickard 2006 (Postnatal Surgery)[53]	• Angular appearance of ventricle 5/17 (29%)
	Sweeny 2012 (Postnatal Surgery)[54]	• Triventricular hydrocephalus 19/33 (57.6%)<br>• Lateral ventricular dilatation or colpocephaly 14/33 (42.4%)
	Nagaraj 2017 (Postnatal Surgery)[39]	• Lateral and third ventricle increased 70/74 (94.6%)
	Danzer 2007 (Postnatal Surgery and Fetal Open Surgery)[40]	• Antero-Posterior (AP) diameter of the fourth ventricle of MMC patients (who were operated on

		postnatally and prenatally) is significantly smaller than controls $p \leq 0.001$
Cortical Abnormalities	Sival 2011 (Postnatal Surgery)[62]	• Diffuse white matter oedema 33/40 (82.5%)<br>• Grey matter oedema 30/40 (75%)
	Danzer 2007 (Postnatal Surgery and Fetal Open Surgery)[40]	• Brain thickness reduced pre and postnatally in MMC patients having postnatal surgery, and MMC patients having Fetal open surgery $p \leq 0.0001$
	Rethman 2017 (Fetal Open Surgery)[42]	• Polymicrogyria 1/27 (3.7%)
Subarachnoid Spaces and Cerebro-Spinal Fluid (CSF) Abnormalities	Danzer 2007 (Postnatal Surgery)[40]	• The CSF space at the level of the cerebellar width ($p \leq 0.0001$) and AP diameter of cerebellum ($p \leq 0.001$) was reduced prenatally and postnatally in MMC
	Rethman 2017 (Fetal Open Surgery)[42]	• The total subarachnoid space at the level of the temporal horns was smaller in MMC and myeloschisis (MS) patients 27/27 (100%)<br>• Supratentorial spaces were smaller in MS patients 9/27 (33.3%)
	Danzer 2007 (Fetal Open Surgery)[40]	• No supratentorial CSF in the subarachnoid space was seen prior to surgery 22/22 (100%)
	Nagaraj 2017 (Postnatal and Fetal Open Surgery)[39]	• Effacement of extra-axial CSF in all MMC patients prenatally was 94/102 (92.1%) in the antenatal period, and 23/102 (22.5%) in the postnatal period

Table 8: Additional supratentorial intracranial findings using traditional MRI sequences

**Reported Abnormality**	**First Author, Year of Publication and timing of MMC surgery**	**Description n/N (%) or *p* value**
Intracranial Haemorrhages and cysts	Mangels 2000 (Fetal Open Surgery)[17]	• Periventricular Leukomalacia 3/4 (75%)<br>• Subependymal haemorrhage 1/4 (25%)<br>• Subdural haematoma 1/4 (25%)<br>• Lobar haemorrhage 1/4 (25%)
Signal Intensity Ratio Changes	Rangasami 2012 (Postnatal Surgery)[61]	• Signal intensity ratio is higher in the cerebellar vermis and periventricular region of MMC patients 15/15 (100%)

Table 9: Miscellaneous intracranial findings using traditional MRI sequences

Reported Feature	First Author, Year of Publication and timing of MMC surgery	Description n/N (%) or *p* value
Posterior Fossae Measurements	Aertsen 2019 (Postnatal and Fetal Open surgery)[6]	• In MMC patients who had Fetal open surgery TCD, CSO, TDPF, PFA showed significant differences within 1 week of prenatal surgery (p 0.01 to ≤ 0.001), most evident of which is cerebellar herniation level (CHL), and CSO angle (p ≤ 0.0001)
	Rethmann 2017 (Fetal Open surgery)[42]	• CSO angle increased significantly between pre and post-operative MRI 27/27 (100%)<br>• The cerebral bony BPD increased significantly at postoperative MRI 27/27 (100%)<br>• Posterior fossae volume (PFV), PFA, and TDPF were still smaller in MMC than in controls after surgery 27/27 (100%)
	Grant 2011 (Postnatal and Fetal Open surgery)[36]	• CSO angle starts off as acute (p≤0.001) but becomes similar to controls after birth in all MMC patients. Antenatal repair had little effect.<br>• At birth the posterior fossae size for MMC patients with postnatal repair was reduced more than MMC patients who had antenatal repair and controls p=0.015
	Nagaraj 2020 (Fetal Open and Fetoscopic Surgery)[45]	• Improvement in posterior fossae crowding was seen in MMC patients after open Fetal surgery 5/5 (100%), and fetoscopic surgery 6/6 (100%)
**Anatomical Descriptive Markers of Chiairi II Malformation**	Rethmann 2017 (Fetal Open surgery)[42]	• Persistent herniation of the choroid plexus through the foramen magnum was seen in cases of reversed Chiari II 12/27 (44.4%)<br>• Flattening of the inferior pontine notch was present after birth with resolved hindbrain herniation 21/27 (77.8%)<br>• The tectal plate remained dysplastic in all neonates with resolved hindbrain herniation 25/27 (92.6%)
**Subarachnoid space and CSF**	Mehollin-Ray 2014 (Fetal Open surgery)[44]	• MMC patients showed an increase in extra-axial space of 5.5 mm on average after surgery 10/12 (83.3%)

	Rethmann 2017 (Fetal Open surgery)[42]	• Supratentorial total subarachnoid space widened postoperatively 22/25 (88%)<br>• Post-operatively the total subarachnoid spaces increased more in myeloschisis than in MMC 9/27 (33.3%)<br>• In all cases of resolved hindbrain herniation, total subarachnoid space decreased 1-3 mm width postnatally 25/25 100%
	Danzer 2007 (Postnatal and Fetal open surgery)[40]	• Subarachnoid space and supratentorial CSF remained reduced postnatally in MMC patients who had postnatal surgery $p \leq 0.0001$<br>• Subarachnoid space and supratentorial CSF improved in MMC patients who had prenatal surgery<br>• In MMC patients who had prenatal surgery, the CSF space at the level of the cerebellar width ($p \leq 0.0001$) and AP diameter of the cerebellum ($p \leq 0.001$) reduced and improved postnatally
	Nagaraj 2020 (Fetal Open and Fetoscopic Surgery)[45]	• Patency of extra-axial space was seen in MMC patients after open Fetal surgery 5/5 (100%), and fetoscopic surgery 6/6 (100%)
**Ventricular system measurements**	Nasiadko 2014 (Fetal Open Surgery)[25]	• Resolved aqueductal stenosis post-surgery 7/9 (77.8%)
	Rethmann 2017 (Fetal Open surgery)[42]	• Ventricular width index (VWI) increased in 2/27 (7.4%), and decreased in 12/27 (44.4%) antenatally<br>• VWI increased in 3/27 (11.1%) and decreased in 10/27 (37%) in the neonatal period.
	Nagaraj 2017 (Postnatal and Fetal Open Surgery)[39]	• The prenatal lateral and third ventricle size was significantly smaller than the postnatal ventricular size in both the prenatal and postnatal repair groups $p \leq 0.001$

Table 10: Additional post-operative MRI features using traditional MRI sequences

Reported Abnormality	First Author, Year of Publication and timing of MMC surgery	Intracranial Location and Description n/N (%) or p value reported in individual studies
	Mignone Philpott 2013 (Postnatal Surgery)[47]	Infratentorial:

Apparent Diffusion Co-efficient (ADC) value changes in MMC brains		• ADC values in MMC cerebella higher than in controls *p*=0.0126
	Shrot 2006 (Postnatal Surgery)[49]	Supratentorial:<br>• ADC values lower in frontal (*p*=0.01) and temporal lobes (*p*=0.05) in MMC brains
	Sanz-Cortes 2020 (Fetal Open and Fetoscopic Surgery)[48]	Supratentorial:<br>• ADC values were higher in the genu of the corpus callosum in MMC undergoing open Fetal surgery 16/30 (53.3%)<br>• ADC values were higher in the genu of the corpus callosum in MMC undergoing fetoscopic Fetal surgery 14/27 (51.9%)<br>• ADC values where higher in basal ganglia of MMC undergoing open Fetal surgery 16/30 (53.3%)<br>• ADC values were higher in basal ganglia of MMC undergoing fetoscopic surgery 14/27 (51.9%)
Fractional Anisotropy Changes	Woitek 2016 (Postnatal Surgery)[50]	Fractional Anisotropy significantly higher in the midbrain of MMC patients p=0.003
Abnormal high T2 signal	Warner 2019 (Postnatal Surgery)[8]	Infratentorial:<br>• Abnormal high T2 signal in cerebellar vermis 10/20 (50%) at 20-22 weeks, and 11/20 (55%) at 30-32 weeks.
Ventricular Abnormalities	Sival 2011 (Postnatal Surgery)[62]	Supratentorial:<br>• Aqueductal stenosis 38/40 (95%)<br>• Occipital horn enlargement 40/40 (100%)<br>• Temporal horn enlargement 22/40 (55%)
	Zarutskie 2019 (Fetal Open and Fetoscopic Surgery)[51]	Supratentorial:

		• Increased ventricular volume and increased ventricular volume growth post open and fetoscopic Fetal surgery 16/48 (33.3%)
Intraventricular and Intracranial haemorrhages	Nagaraj 2020 (Fetal Open and Fetoscopic Surgery)[45]	Miscellaneous:<br>• Intraventricular haemorrhage in MMC brain of open Fetal surgery 1/47 (2.1%)<br>• Intracranial haemorrhage in MMC brains of open Fetal surgery 10/47 (21.3%)<br>• Intraventricular haemorrhage in MMC brain of fetoscopic Fetal surgery 1/15 (6.7%)<br>• Intracranial haemorrhage in MMC brains of fetoscopic Fetal surgery 3/15 (20%)

Table 11: Additional intracranial findings using advanced MRI sequences
