# Supplementary material for: What brain abnormalities can magnetic resonance imaging detect in foetal and early neonatal spina bifida: a systematic review
Source: Neuroradiology. 2021 Nov 18;64(2):233–45. doi: 10.1007/s00234-021-02853-1 (PMC8789702; doi:10.1007/s00234-021-02853-1)
Supplement: Supplementary file 2 — Supplementary file2 (PDF 62 KB) [file 234_2021_2853_MOESM2_ESM.pdf]

Online Resource: Search Strategy

1. Fetus/
2. Infant, Newborn/
3. f?etus*.tw.
4. newborn*.tw.
5. neonate*.tw.
6. prenatal.tw.
7. antenatal.tw.
8. (in adj3 utero).tw.
9. or/1-8
10. neural tube defects/
11. anencephaly/
12. arnold-chiari malformation/
13. encephalocele/
14. meningocele/
15. meningomyelocele/
16. spinal dysraphism/
17. spina bifida cystica/
18. spina bifida occulta/
19. (neural adj3 tube defect*).tw.
20. anencephaly.tw.
21. (Arnold-chiari adj3 malformation).tw.
22. encephalocele*.tw.
23. meningocele.tw.
24. myelomeningocele.tw.
25. meningomyelocele.tw.
26. (spin* adj3 dysraph*).tw.
27. spina bifida cystica.tw.
28. spina bifida aperta.tw.
29. spina bifida occulta.tw.
30. (open adj3 spina bifida).tw.
31. (closed adj3 spina bifida).tw.
32. myeloschisis.tw.
33. myelocele.tw.
34. acrania.tw.

35. NTD.tw.

36. MMC.tw.

37. chiari II.tw.

38. or/10-37

39. magnetic resonance imaging/

40. diffusion magnetic resonance imaging/

41. echo-planar imaging/

42. fluorine-19 magnetic resonance imaging/

43. diffusion tensor imaging/

44. functional neuroimaging/

45. brain mapping/

46. Imaging, Three-Dimensional/

47. Magnetic Resonance Imaging, Cine/

48. magnetic resonance imag*.tw.

49. (echo planner adj3 imag*).tw.

50. relaxometry.tw.

51. diffusion tensor imag*.tw.

52. tractography.tw.

53. diffusion weighted imag*.tw.

54. (MRI adj3 3D reconstruction).tw.

55. (MRI adj3 super resolution).tw.

56. t2*.tw.

57. t1.tw.

58. perfusion ASL.tw.

59. phase contrast.tw.

60. spectroscopy.tw.

61. MRI.tw.

62. BOLD Imag*.tw.

63. ultrasonography, doppler/

64. ultrasonography, doppler, duplex/

65. ultrasonography, doppler, color/

66. ultrasonography, doppler, transcranial/

67. ultrasonography, prenatal/

68. Ultrasonography/

69. Imaging, Three-Dimensional/

70. Ultrasonics/

71. ultrasound.tw.

72. ultrasonograph*.tw.

73. sonograph*.tw.

74. (2d adj3 ultrasound).tw.

75. (3d adj3 ultrasound).tw.

76. (4d adj3 ultrasound).tw.

77. (2 dimensional adj3 ultrasound).tw.

78. (two dimensional adj3 ultrasound).tw.

79. (three dimensional adj3 ultrasound).tw.

80. (3 dimensional adj3 ultrasound).tw.

81. (four dimensional adj3 ultrasound).tw.

82. (4 dimensional adj3 ultrasound).tw.

83. ultrasonics.tw.

84. or/39-83

85. brain/

86. brain stem/

87. cerebral ventricles/

88. gray matter/

89. white matter/

90. spinal cord/

91. cervical cord/

92. cerebrum/

93. basal ganglia/

94. cerebral cortex/

95. "septum of brain"/

96. septum pellucidum/

97. telencephalic commissures/

98. anterior cerebellar commissure/

99. corpus callosum/

100. white matter/

101. cerebellum/

102. cerebellar cortex/

103. cerebellar vermis/

104. cerebellar nuclei/

105. cerebellopontine angle/

106. pons/

107. Cisterna Magna/

108. Cranial Fossa, Posterior/

109. spine/

110. lumbar vertebrae/

111. sacrum/

112. thoracic vertebrae/

113. (brain adj3 abnormal*).tw.

114. (brain adj3 anomal*).tw.

115. brain stem.tw.

116. cerebral ventricle*.tw.

117. cerebrum.tw.

118. cerebral cortex.tw.

119. anterior cerebellar commissure.tw.

120. corpus callosum.tw.

121. (septum adj3 brain).tw.

122. cerebellum.tw.

123. cerebellar cortex.tw.

124. cerebellar nucle*.tw.

125. cerebellopontine angle.tw.

126. pons.tw.

127. white matter.tw.

128. gray matter.tw.

129. cisterna mag*.tw.

130. septum pellucidum.tw.

131. skull base.tw.

132. (cranial adj3 posterior fossa?).tw.

133. lumbar vertebra?.tw.

134. thoracic vertebra?.tw.

135. sacral.tw.

136. ventricle.tw.

137. ventriculomegaly.tw.

138. (cavum adj3 septum pellucid*).tw.

139. CSP.tw.

140. or/85-139

141. 9 and 38 and 84 and 140

145. or/39-42

146. 9 and 38 and 145 and 140

Key:

/ = Indexing term (MESH heading)

tw = Text word search in title or abstract

adj3 = Terms within three words of each other

* = Truncation

? = Used to represent a single character when there are variable spellings of a word

141 = Search for brain abnormalities detected by magnetic resonance imaging compared to ultrasound in fetal and early neonatal (<28 days) spina bifida

146 = Search for brain abnormalities detected by magnetic resonance imaging in fetal and early neonatal spina bifida
